# Single-Cell Insights into Medicinal Plant Development and Metabolism

**DOI:** 10.3390/plants15182799

**Published:** 2026-09-12

**Authors:** Baoping Jiang, Liang Le

**Affiliations:** 1 Beijing Life Science Academy, Beijing 102200, China; bpjiang@implad.ac.cn; 2 State Key Laboratory for Quality Ensurance and Sustainable Use of Dao-di Herbs, Institute of Medicinal Plant Development, Chinese Academy of Medical Sciences & Peking Union Medical College, Beijing 100193, China; 3 Biotechnology Research Institute, Chinese Academy of Agricultural Sciences, 12 Zhongguancun South Street, Beijing 100081, China

**Keywords:** artificial intelligence, medicinal plants, single-cell RNA-seq, scATAC-seq, spatial metabolomics, specialized metabolism

## Abstract

Medicinal plants are major sources of therapeutic natural products, yet the cell-type-specific organization that governs metabolite biosynthesis, transport, and storage remains imperfectly resolved by organ-level omics. This review synthesizes studies published up to June 2026 that used single-cell, single-nucleus, spatial, metabolomic, and epigenomic approaches to medicinal plant systems, following a PRISMA-guided literature search across PubMed, Web of Science, Scopus, and CNKI. Emerging evidence shows that specialized metabolism is organized through discrete and often rare cell populations, including idioblasts, laticifers, glandular trichomes, secretory epidermal cells, internal phloem-associated parenchyma, cork and periderm cells, mesophyll cells, and other biosynthetic niches. Single-cell RNA sequencing has defined these populations and reconstructed developmental trajectories, whereas single-cell metabolomics and mass spectrometry imaging reveal that metabolite accumulation frequently diverges from biosynthetic gene expression because of intercellular transport, storage capacity, and subcellular compartmentation. Single-cell ATAC-seq and multiome profiling further identify cell-type-specific regulatory regions, transcription factors, and candidate promoters controlling metabolic competence. Together, these technologies are reshaping medicinal plant biology from pathway-centric catalogs into spatially and developmentally resolved cellular maps. We highlight how artificial intelligence (AI)-assisted integration can accelerate cell annotation, regulatory network inference, metabolite assignment, and prioritization of biosynthetic genes, transporters, and engineering targets. Future progress will depend on comparative medicinal plant atlases, improved recovery of recalcitrant tissues, matched transcriptomic, metabolomic, and spatial designs, and functional validation of cell-type-specific mechanisms.

## 1. Introduction

Medicinal plants are major sources of chemically diverse natural products [1,2]. Many clinically important compounds and pharmacologically active scaffolds originate from specialized plant metabolism, including vinblastine and vincristine from *Catharanthus roseus*, paclitaxel from *Taxus* species, camptothecin from *Camptotheca acuminata* and *Ophiorrhiza pumila*, artemisinin from *Artemisia annua*, ginsenosides from *Panax* species, tanshinones from *Salvia miltiorrhiza*, hyperforin from *Hypericum perforatum*, and patchouli alcohol from *Pogostemon cablin* [3,4,5,6]. Other medicinal plants produce abundant saponins, phenylethanoid glycosides, flavonoids, phenolic acids, polysaccharides, and volatile terpenes [7,8]. These metabolites are valuable not only as drugs and lead compounds, but also as ecological mediators that contribute to defense, attraction, stress tolerance, allelopathy, and plant–microbe interactions [9,10,11,12,13].

Genome sequencing, transcriptomics, metabolomics, and synthetic biology have greatly advanced medicinal plant research. Even so, the cellular organization of specialized metabolism remains incompletely understood [14,15,16,17,18,19,20]. One reason is that most omics studies still rely on bulk tissues or whole organs. Bulk RNA-seq can identify candidate pathway genes through co-expression with known enzymes, but this strategy becomes less reliable when pathway modules are split among different cell types, when genes are not co-regulated, or when rare biosynthetic cells are masked by abundant neighboring cells [21,22,23,24]. Bulk metabolomics can measure active compounds, but it cannot determine whether a metabolite is synthesized, imported, stored, or degraded in a given cell type. Imaging mass spectrometry can localize metabolites, but without cell-type-resolved transcriptomic information, it often cannot identify the enzymes or regulators that shape the observed chemical pattern [25,26,27,28]. These limitations are particularly important in medicinal plants, where bioactive compounds often accumulate in rare secretory cells, glandular trichomes, laticifers, idioblasts, bark or cork layers, root storage cells, and developmentally restricted tissues.

The idea that specialized plant metabolism is cell-type-specific has a long history. In *Catharanthus roseus*, classical studies showed that monoterpene indole alkaloid (MIA) biosynthesis is distributed across internal phloem-associated parenchyma (IPAP), epidermal cells, idioblasts, and laticifers [29,30,31,32,33]. In *Artemisia annua*, artemisinin biosynthesis is associated with glandular secretory trichomes [34,35,36]. In opium poppy, benzylisoquinoline alkaloid biosynthesis involves companion cells, sieve elements, and laticifers [37,38]. These studies established the conceptual basis for cell-type-specific metabolism, but they relied mainly on RNA in situ hybridization, immunolocalization, laser microdissection, reporter gene analysis, or tissue-specific extraction. As a result, they could not provide genome-wide views at single-cell resolution.

Single-cell omics is now changing this landscape. Single-cell RNA sequencing (scRNA-seq) and single-nucleus RNA sequencing (snRNA-seq) can identify cell types, infer developmental trajectories, and map pathway gene expression across thousands of individual cells [39,40,41,42]. Single-cell metabolomics adds a complementary layer by measuring metabolites in individual cells, making it possible to test whether metabolite accumulation matches biosynthetic gene expression [25,43,44,45,46]. Single-cell ATAC-seq (scATAC-seq) and scRNA-seq multiome profiling further reveal accessible chromatin regions and transcription factor motifs that may regulate cell-type-specific metabolism [47,48,49,50]. Spatial transcriptomics and mass spectrometry imaging (MSI) then place these single-cell signals back into intact tissues, allowing biosynthetic niches to be interpreted in their spatial context [26,51,52,53,54].

Medicinal plant research has therefore moved beyond isolated examples toward a broader cell-resolved framework. *Catharanthus roseus* has become an important model for studying the multicellular compartmentalization of monoterpene indole alkaloid metabolism [25,33,48]. In *Taxus*, single-nucleus perturbation strategies have helped identify missing paclitaxel biosynthetic genes and support heterologous pathway reconstruction [55,56,57]. In *Hypericum perforatum*, scRNA-seq identified Hyper cells and contributed to completion of the hyperforin pathway [58]. Studies in *Panax* have linked ginsenoside biosynthesis with root cell differentiation and spatial metabolite distribution [59,60]. Work in *Artemisia* [61], *Nepeta tenuifolia* [62], *Salvia* [63], *Ophiorrhiza* [64], *Camptotheca* [65], *Cistanche* [66], *Andrographis* [67], *Gynostemma* [68], *Pogostemon* [69], and *Dendrobium* [70] further shows that medicinal plants organize specialized metabolism according to distinct cellular architectures.

In this review, we examine how single-cell omics is being used to study medicinal plants. We first introduce the main single-cell and spatial technologies currently applied in this field. We then synthesize the specialized cell types identified by these approaches. Next, we discuss mechanistic insights from single-cell transcriptomics, metabolomics, and chromatin accessibility, with emphasis on metabolic compartmentalization, rare biosynthetic cells, cell-to-cell transport, developmental regulation, and transcriptional control. Finally, we consider future directions, particularly the use of artificial intelligence to integrate single-cell multi-omics data and accelerate medicinal plant metabolic discovery.

## 2. Review Methodology

**Figure 1:**
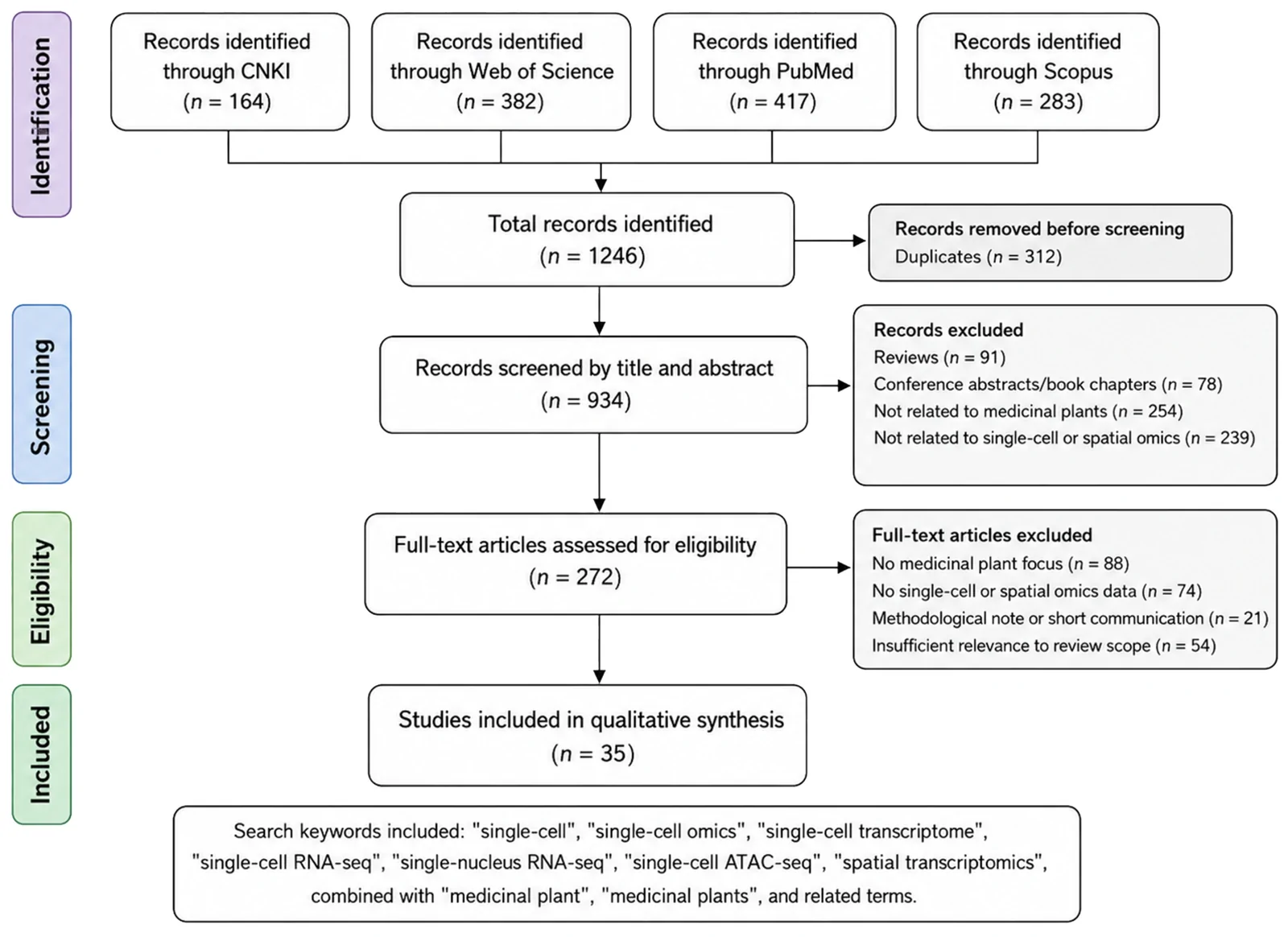
PRISMA-guided literature search and study-selection workflow. The flow chart reports database-specific retrieval, duplicate removal, title-and-abstract screening, full-text eligibility assessment, exclusion categories, and the 35 primary studies retained for qualitative synthesis. Searches were completed on 16 June 2026; detailed search terms and eligibility criteria are provided in Section 2.

A comprehensive literature review was conducted following the PRISMA guidelines, a multi-step systematic framework for identifying and selecting relevant studies [71]. The process comprised three primary stages: (1) Identification, (2) Screening, and (3) Inclusion. Literature searches were carried out in four bibliographic databases: China National Knowledge Infrastructure (CNKI), Web of Science (WoS) Core Collection, PubMed, and Scopus. To ensure broad coverage, the “Topic” field was used in CNKI and WoS, while the “All Fields” option was used in PubMed. The final search was conducted on 16 June 2026. Search strings were adapted to each database and combined (“single-cell” OR “single-cell omics” OR “single-cell transcriptome” OR “single-cell RNA-seq” OR “single-nucleus RNA-seq” OR “single-cell ATAC-seq” OR “spatial transcriptomics”) with (“medicinal plant” OR “medicinal plants”) using Boolean operators. This approach retrieved 164 records from CNKI, 382 from WoS, 417 from PubMed, and 283 from Scopus. After 312 duplicates were removed, 934 records were screened by title and abstract. Title-and-abstract screening left 272 articles for full-text eligibility assessment, and 35 primary studies were retained for qualitative synthesis, as illustrated in Figure 1.

Eligibility was assessed in two stages. Title-and-abstract screening excluded reviews (*n* = 91), conference abstracts or book chapters (*n* = 78), records unrelated to medicinal plants (*n* = 254), and records unrelated to single-cell or spatial omics (*n* = 239), leaving 272 articles for full-text assessment. Full-text exclusions comprised studies without a medicinal-plant focus (*n* = 88), studies without single-cell or spatial-omics data (*n* = 74), methodological notes or short communications (*n* = 21), and studies with insufficient relevance to the review’s cell-resolved mechanistic scope (*n* = 54). The final qualitative synthesis therefore included 35 primary studies that directly examined medicinal plants using single-cell, single-nucleus, spatial-transcriptomic, or spatial/single-cell-metabolomic approaches. CNKI was retained to reduce language and regional coverage bias; all CNKI records were assessed using the same eligibility criteria as records from the international databases. The complete screening logic and counts are shown in Figure 1.

## 3. Single-Cell and Spatial Omics Technologies in Medicinal Plants

### 3.1. ScRNA-seq

**Figure 2:**
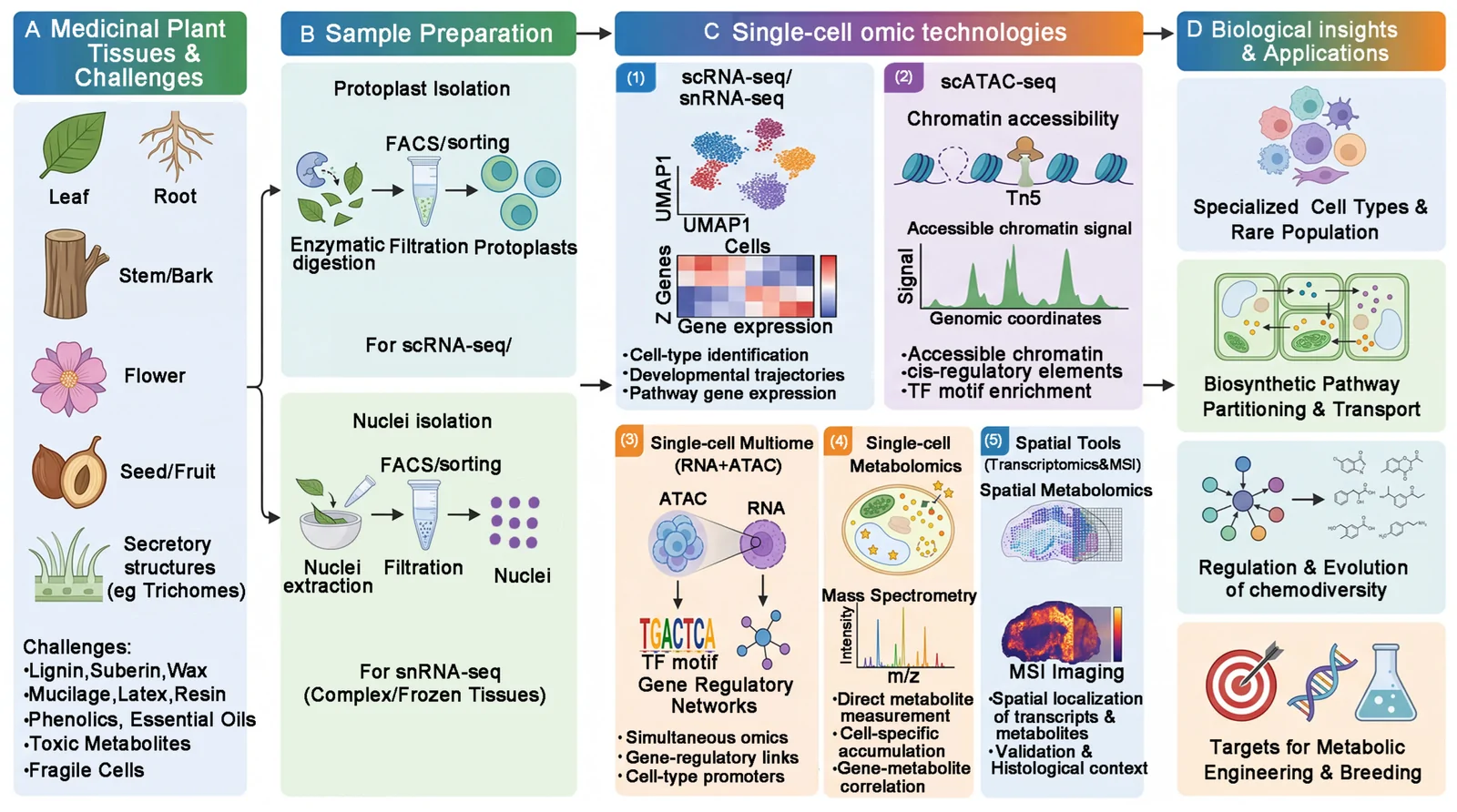
Single-cell omics technologies used in medicinal plant research. (**A**). Medicinal plant tissues, including leaves, roots, stems/bark, flowers, seeds/fruits, and specialized secretory structures, can be processed through protoplast isolation or nuclei isolation for downstream single-cell analyses. (**B**). Protoplast-based workflows generally involve enzymatic digestion, filtration, fluorescence-activated cell sorting (FACS) or enrichment, and quality control, whereas nuclei-based workflows enable profiling of tissues that are difficult to dissociate into intact cells. (**C**). Major single-cell omics technologies include single-cell or single-nucleus RNA sequencing (scRNA-seq/snRNA-seq) for cell-type identification, developmental trajectory inference, and pathway gene expression analysis; single-cell ATAC sequencing (scATAC-seq) for chromatin accessibility and *cis*-regulatory element discovery; single-cell metabolomics for metabolite localization and gene–metabolite association; and multiome integration for linking transcriptomic and epigenomic states. Spatial transcriptomics, RNA in situ hybridization, imaging mass spectrometry, and microscopy provide tissue-context validation. (**D**). These approaches reveal specialized cell types, biosynthetic partitioning, metabolite transport, chemodiversity evolution, and candidate targets for medicinal plant breeding and synthetic biology.

ScRNA-seq is the most widely used single-cell approach in medicinal plant research. Most studies generate protoplasts by enzymatic digestion and then use either droplet-based platforms, such as 10× Genomics, or plate-based full-length transcriptome methods, such as SMART-seq [72,73,74]. In medicinal plants, scRNA-seq has been applied to leaves, roots, stems, shoot apices, flowers, somatic embryos, glandular-trichome-containing tissues, and root tips. These datasets have helped identify major cell types, rare cell populations, cell-type-specific markers, developmental trajectories, and specialized metabolic programs (Figure 2, Table 1).

A major advantage of scRNA-seq is its ability to resolve cellular heterogeneity. In *Catharanthus roseus*, leaf scRNA-seq produced a high-resolution atlas of more than 30,000 cells and revealed sequential partitioning of the MIA pathway among IPAP cells, epidermal cells, and idioblasts [44]. In *Camptotheca acuminata*, scRNA-seq of shoot apices and leaves mapped the spatiotemporal distribution of camptothecin pathway genes and suggested contributions from proliferating and vascular cells [65]. In *Panax* root tips, scRNA-seq reconstructed endodermal differentiation and revealed epidermis-specific expression of key ginsenoside genes [60]. In *Artemisia argyi*, scRNA-seq identified a glandular-trichome-associated epidermal subcluster and guided the discovery of sesquiterpene synthases [75]. In *Pogostemon cablin*, scRNA-seq of globular and cotyledonary somatic embryos identified PcNAC048 as a dual regulator of lateral root development and patchouli alcohol biosynthesis [69].

Protoplast-based scRNA-seq also has clear limitations in medicinal plants. Many medicinal tissues contain lignin, suberin, wax, mucilage, latex, resin, phenolics, essential oils, or toxic metabolites, all of which can interfere with cell wall digestion and cell viability. Fragile specialized cells, including laticifers, mature glandular trichomes, resin ducts, oil cells, cork cells, and xylem-associated cells, may be poorly recovered. Enzymatic digestion can also induce stress-responsive genes and alter metabolic pathway expression [45,76]. Therefore, scRNA-seq studies in medicinal plants require careful optimization, short digestion times, viability checks, marker validation, and parallel spatial or histological evidence (Figure 2).

### 3.2. SnRNA-seq

SnRNA-seq offers an alternative when protoplast isolation is inefficient or biased. Rather than digesting intact cells, snRNA-seq isolates nuclei from fresh or frozen tissues. This reduces biases caused by cell wall digestion and makes it possible to analyze mature, woody, waxy, or metabolite-rich tissues [77,78,79,80]. The approach is particularly useful for medicinal plants with complex tissues, including roots, bark, needles, periderm, glandular trichomes, and mature leaves. snRNA-seq has already been used in *Taxus*, *Artemisia annua*, *Panax ginseng*, and other medicinal plants. In *Artemisia annua*, single-nucleus and spatial transcriptomics resolved glandular secretory trichome development and localized artemisinin biosynthesis to secretory cells within the 10-cell glandular trichome structure [61]. In *Panax ginseng*, snRNA-seq combined with MALDI-MSI was used to map the multicellular compartmentalization of ginsenosides in leaves, stems, and roots [59]. In *Taxus*, multiplexed perturbation single-nucleus profiling improved co-expression resolution and enabled the discovery of missing paclitaxel genes [55]. The main limitation of snRNA-seq is that nuclear RNA reflects nascent or retained transcripts and may not fully match cytoplasmic mRNA abundance. Some metabolic genes may be weakly represented in nuclei, so direct comparisons between scRNA-seq and snRNA-seq should be made cautiously. Even with this limitation, snRNA-seq is likely to become a preferred option for many medicinal plants because it is compatible with frozen samples, difficult tissues, and rare cell types.

Compared with scRNA-seq, snRNA-seq generally provides lower cytoplasmic and organellar transcript coverage because it enriches nascent, unspliced, and nucleus-retained RNA. This difference is consequential for pathway reconstruction: transcripts encoding enzymes that act in plastids, mitochondria, the cytosol, the endoplasmic reticulum, or vacuolar transport routes may be underrepresented even when their genes are transcriptionally active. Conversely, intronic reads can improve detection of active transcription and regulatory state. Pathway inference from snRNA-seq should therefore combine exon- and intron-aware quantification, avoid interpreting undetected transcripts as biological absence, and validate key pathway steps with matched scRNA-seq, tissue RNA-seq, spatial transcriptomics, in situ assays, or metabolite localization whenever feasible.

### 3.3. ScATAC-seq and Single-Cell Multiome

ScATAC-seq maps chromatin accessibility at single-cell resolution, revealing regulatory regions, transcription factor motifs, and cell-type-specific *cis*-regulatory landscapes [49,81,82]. Its application in medicinal plants is still at an early stage, but its rationale is compelling. Specialized metabolism is often controlled by cell-type-specific transcription factors, promoter regions, and chromatin states. ScATAC-seq can therefore help explain why pathway genes are activated in particular cell types.

The clearest example to date comes from *Catharanthus roseus*. Single-cell RNA/ATAC multiome profiling of mature leaves reconstructed the MIA regulatory landscape and identified idioblast-specific accessible chromatin regions enriched for MYB and WRKY motifs [48]. Integration with DAP-seq and functional assays further identified CrIDM1 (Idioblast MYB1) as an idioblast-specific MYB transcription factor that activates late MIA genes, including Deacetoxyvindoline 4-hydroxylase (D4H) and Deacetylvindoline 4-O-acetyltransferase (DAT) [48]. In *Camptotheca acuminata*, single-nucleus multiome analysis identified a rare Strictosidine Synthase (STR)-positive cell population and STR+ cell-specific accessible chromatin enriched for MYB motifs. This finding suggests that rare camptothecin biosynthetic cells are defined by both transcriptional and chromatin states [83].

Single-cell multiome approaches are especially useful because RNA and ATAC signals are obtained from the same nucleus [83]. This allows cell identity, gene expression, chromatin accessibility, and regulatory motif enrichment to be linked directly. In future studies, multiome datasets may help identify cell-type-specific promoters for metabolic engineering, predict transcription factors that control rare biosynthetic cells, and reveal how medicinal pathways have evolved at the regulatory level.

### 3.4. Single-Cell Metabolomics

Single-cell metabolomics is particularly important for medicinal plant research because it measures metabolites directly in individual cells. Transcriptomics can indicate biosynthetic potential, but it cannot show where metabolites actually accumulate. A compound may be synthesized in one cell, stored in another, transported across tissues, or sequestered in vacuoles, cuticles, resin ducts, or secretory cavities [25,46].

Early studies in *Catharanthus roseus* used imaging mass spectrometry and live MSI to show that catharanthine, ajmalicine, serpentine, strictosidine, and related monoterpene indole alkaloids (MIAs) accumulate mainly in idioblasts and laticifers, whereas secologanin is enriched in epidermal cells [25]. Later single-cell metabolomics of *Catharanthus* leaves showed that alkaloid localization is complex and developmentally dynamic [43]. Quantitative plate-based single-cell mass spectrometry then measured 16 natural products across leaf, root, and petal cells. This work showed that many compounds reach millimolar concentrations and that metabolite localization can vary by organ and genotype [45]. More recently, same-cell single-cell metabolome and transcriptome profiling enabled direct gene–metabolite correlation within individual plant cells [46].

Despite its value, single-cell metabolomics remains technically demanding. It requires sensitive mass spectrometry, careful cell isolation, metabolite standards, rapid sample handling, and computational pipelines for peak detection and annotation. Throughput is still lower than that of scRNA-seq, and metabolite coverage remains limited. Even so, single-cell metabolomics provides direct chemical evidence that cannot be obtained from transcriptomic approaches alone.

### 3.5. Spatial Metabolomics and Spatial Transcriptomics

MSI is currently one of the most useful spatial technologies in medicinal plant research. Matrix-assisted laser desorption/ionization MSI (MALDI-MSI), atmospheric-pressure MALDI-MSI (AP-MALDI-MSI), MALDI-2-MSI, desorption electrospray ionization MSI (DESI-MSI), and related platforms have been used to map alkaloids, terpenoids, saponins, phenolics, and pigments in plant tissues [7,8,51,84,85]. For instance, AP-MALDI-MSI showed that tanshinones accumulate in the outer cork layer of mature roots, supporting scRNA-seq evidence that cork cells are biosynthetically active in *Salvia miltiorrhiza* [63]. In *Panax*, MALDI-2-MSI mapped ginsenoside intermediates and final products to root epidermal and vascular-associated regions [60]. In *Andrographis paniculata*, DESI-MSI revealed that andrographolide-type diterpenoids accumulate in non-veinal leaf regions and outer stem tissues [67]. In *Taxus*, MSI has helped connect taxane distribution with stem and leaf tissue organization [86,87].

Spatial transcriptomics is at an earlier stage in medicinal plants but holds considerable promise. In *Artemisia annua*, spatial transcriptomics validated glandular secretory trichome regions and supported the localization of artemisinin pathway gene expression [61]. Future plant spatial transcriptomics will need improved tissue fixation, sectioning, RNA capture, cell wall permeabilization, and integration with histology and MSI. The long-term goal is to combine scRNA-seq, scATAC-seq, spatial transcriptomics, and spatial metabolomics into a unified tissue-scale cell atlas.

## 4. Specialized Cell Types Revealed in Medicinal Plants

**Figure 3:**
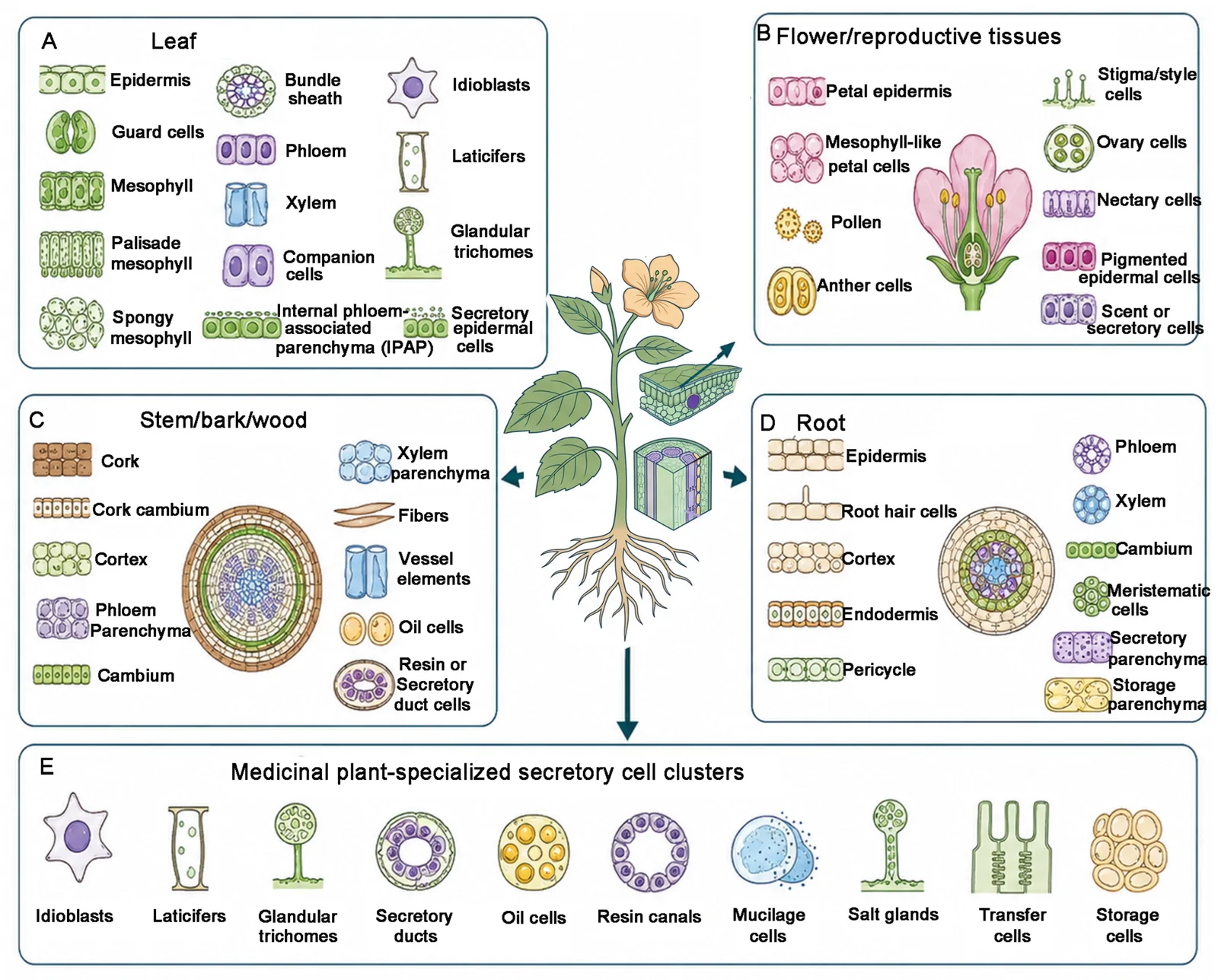
Cell-type atlas of medicinal plants revealed by single-cell omics. (**A**–**D**). Major cell populations identified across leaves (**A**), flowers (**B**), stems (**C**), roots (**D**), and reproductive tissues are summarized together with medicinal-plant-specific secretory and storage cells. (**E**). Frequently reported specialized groups include idioblasts, laticifers, glandular trichomes, secretory epidermal cells, phloem-associated parenchyma, cambium-associated cells, petal secretory cells, and root storage cells.

Most medicinal-plant atlases first recover conserved cell classes shared with non-medicinal plants, including epidermal, guard, mesophyll, vascular, cambial, endodermal, pericycle, and storage-parenchyma populations. These common classes provide anatomical landmarks for annotation and a baseline for cross-species comparison. Medicinal relevance emerges when particular members of these lineages acquire unusual biosynthetic, transport, secretory, or storage states, or when rare structures such as idioblasts, laticifers, and glandular trichomes become dominant metabolic niches. The subsections below therefore begin from this specialized cellular framework and then focus on specialized states revealed by cell-resolved omics (Figure 3; Table 2).

### 4.1. Idioblasts

Idioblasts are one of the most important specialized cell types in medicinal plant single-cell research. In *Catharanthus roseus*, idioblasts accumulate high concentrations of MIAs and express late-pathway genes involved in vindoline biosynthesis and alkaloid storage [25,43,44]. They are rare cells, but they play a central role in late MIA metabolism. scRNA-seq, scMS, and scRNA-ATAC studies show that idioblasts are metabolically active, transcriptionally distinct, and regulated by specific transcription factors such as CrIDM1 [48]. Idioblasts also provide an example of why bulk tissue analysis can be misleading: a small number of cells can dominate the accumulation of pharmacologically important compounds.

Idioblast-like cells or rare alkaloid-associated cells may also exist in other medicinal plants. In *Camptotheca acuminata*, STR+ rare cells identified by single-cell multiome analysis may represent a specialized camptothecin-related cell population [88]. More work is needed to determine whether these cells are anatomically equivalent to idioblasts, epidermal derivatives, vascular-associated cells, or a distinct lineage (Figure 3A, Table 1).

### 4.2. Laticifers

Laticifers are elongated latex-producing cells or cell networks that store defense-related compounds, alkaloids, proteins, and latex-associated metabolites. In *Catharanthus roseus*, live MSI showed that laticifers accumulate MIAs along with idioblasts [25]. However, laticifers are often poorly captured by protoplast-based scRNA-seq because they are long, fragile, and embedded within tissues. This discrepancy illustrates a broader methodological issue: metabolically important cells are not necessarily easy to capture by high-throughput single-cell platforms. Future single-nucleus sequencing, spatial transcriptomics, laser microdissection, and cell-type-specific enrichment will be needed to better characterize laticifers in medicinal plants such as *Catharanthus*, opium poppy, and other latex-producing species.

### 4.3. Internal Phloem-Associated Parenchyma and Phloem Parenchyma

Internal phloem-associated parenchyma is critical in the *Catharanthus* MIA pathway. IPAP cells express upstream methylerythritol phosphate (MEP) and iridoid pathway genes, supplying early monoterpene precursors for downstream alkaloid formation [30,44]. This cell type is central to the IPAP–epidermis–idioblast model of MIA multicellular compartmentalization. Its function is not merely structural; it represents a metabolic source cell from which products must move to epidermal cells.

*Phloem parenchyma* and companion cells also appear in other medicinal pathways. In *Panax*, companion cells and phloem-related populations express selected ginsenoside biosynthetic genes [59,60]. In *Taxus*, vascular and cambium-associated cells are connected with taxane biosynthesis and precursor allocation [55,86]. Phloem-associated cells may therefore function as metabolic hubs, transport interfaces, or developmental sources of specialized metabolites.

### 4.4. Epidermal and Secretory Epidermal Cells

Epidermal cells are repeatedly identified as major metabolic interfaces. In *Catharanthus*, epidermal cells express genes involved in secologanin production, strictosidine formation, and alkaloid scaffold generation [29,44]. In *Ophiorrhiza pumila*, camptothecin biosynthetic genes are preferentially expressed in young epidermal cells, and in situ hybridization confirmed epidermal localization of several known pathway genes [64]. In *Panax*, ginsenosides accumulate in epidermal or cork-like tissues, and pathway genes are distributed among epidermal, guard, palisade, and phloem-related cells [59,60]. In *Camptotheca*, epidermal, vascular, proliferating, and rare STR+ cells may each contribute to different camptothecin pathway steps [65,88].

The epidermis is biologically suited for specialized metabolism because it is exposed to light, herbivores, pathogens, and abiotic stress. It participates in defense, secretion, cuticle formation, volatile emission, and metabolite export. Thus, medicinal plant epidermis should be viewed as an active metabolic interface rather than a passive protective layer.

### 4.5. Glandular Trichomes

Glandular trichomes are specialized secretory organs that synthesize and store terpenoids, phenylpropanoids, flavonoids, and other metabolites. They are key structures in *Artemisia annua*, *Artemisia argyi*, *Nepeta tenuifolia*, *Pogostemon cablin*, and many aromatic medicinal plants [61,69,89].

In *Artemisia annua*, single-nucleus and spatial transcriptomics revealed that artemisinin biosynthesis is concentrated in six secretory cells within the 10-cell glandular secretory trichome structure [61]. In *Artemisia argyi*, glandular trichome metabolomics showed strong enrichment of terpenoids, flavonoids, and fatty acyls; scRNA-seq identified a glandular trichome-associated epidermal subcluster and guided discovery of sesquiterpene synthases such as AarTPS77 [75]. In *Nepeta tenuifolia*, scRNA-seq identified peltate glandular trichome cells and reconstructed their developmental trajectory, linking gland identity to monoterpene biosynthesis [62]. These studies show that glandular trichomes are not uniform secretory sacs but organized multicellular structures with developmental and metabolic specialization.

### 4.6. Cork, Periderm, Cambium, and Bark-Associated Cells

Woody and perennial medicinal plants often accumulate active compounds in bark, cork, cambium, or periderm tissues. These tissues are difficult to analyze because they contain lignified and suberized cell walls, secondary metabolites, and complex developmental gradients (Figure 3C).

In *Salvia miltiorrhiza*, scRNA-seq of young and mature roots, integrated with AP-MALDI-MSI, revealed that tanshinone biosynthesis and accumulation are strongly associated with mature cork cells [63]. A developmental trajectory from pericycle-like cells through phellogen to cork cells connected secondary growth with the acquisition of diterpenoid metabolic competence. In *Taxus*, cambium and bark-like tissues are important for paclitaxel biosynthesis, and single-nucleus perturbation strategies have helped discover missing Taxol genes [55]. In *Cinnamomum camphora*, leaf scRNA-seq identified terpenoid-related gene expression patterns across mesophyll, epidermal-like, epidermal, guard mother, and vascular cells, providing a cellular resource for studying volatile metabolism [90].

**Table 1:** Representative single-cell omics studies in medicinal plants.

Species	Major Metabolites or Pathway	Main Tissue or Organ	Single-Cell or Spatial Modality	Key Contribution	Ref.
*Catharanthus roseus*	Monoterpene indole alkaloids	Leaf, stem, root, petal	scRNA-seq, scMS, scRNA + ATAC, same-cell scMS + scRNA	Established multicellular MIA partitioning and gene-metabolite relationships	[25,33,47,48]
*Taxus* spp.	Paclitaxel and taxanes	Needle, stem, cambium, bark-like tissues	snRNA-seq, perturbation single-nucleus profiling, MSI	Enabled discovery of missing Taxol genes and pathway reconstruction	[86,87]
*Hypericum perforatum*	Hyperforin	Leaf, flower	scRNA-seq, genome-guided pathway reconstruction	Identified Hyper cells and completed hyperforin pathway	[58]
*Camptotheca acuminata*	Camptothecin	Shoot apex, leaf	scRNA-seq, single-cell multiome	Mapped pathway gene distribution and rare STR+ cells	[65,88]
*Ophiorrhiza pumila*	Camptothecin and MIAs	Leaf	scRNA-seq, in situ hybridization, CRISPR	Identified epidermal biosynthesis and OpAVT1-mediated precursor transport	[64]
*Panax ginseng*	Ginsenosides	Root, leaf, stem	scRNA-seq, snRNA-seq, MSI	Revealed multicellular ginsenoside compartmentalization	[59,60]
*Panax notoginseng*/*P. quinquefolium*	Ginsenosides	Root tip	scRNA-seq, MALDI-2-MSI	Compared conserved and divergent root programs	[59,60]
*Artemisia annua*	Artemisinin	Leaf glandular trichome	snRNA-seq, spatial transcriptomics	Resolved GST development and artemisinin-producing cells	[61]
*Artemisia argyi*	Sesquiterpenes and flavonoids	Leaf glandular trichome	scRNA-seq, GT/NGT metabolomics	Identified GT subclusters and terpene synthases	[75]
*Salvia miltiorrhiza*	Tanshinones, phenolic acids	Root	scRNA-seq, AP-MALDI-MSI	Linked mature cork cells to tanshinone biosynthesis	[63]
*Andrographis paniculata*	Andrographolide-type diterpenoids	Leaf, stem	scRNA-seq, DESI-MSI	Connected mesophyll *ApCPS2* expression with light-enhanced diterpenoid accumulation	[67]
*Pogostemon cablin*	Patchouli alcohol	Somatic embryos, leaf	scRNA-seq, functional validation	Identified PcNAC048 as a regulator of root development and patchouli alcohol biosynthesis	[69]
*Cistanche deserticola*	Phenylethanoid glycosides	Succulent stem	scRNA-seq, genome analysis	Linked parasitism-related cells with host adaptation and medicinal metabolism	[66]
*Gynostemma pentaphyllum*	Gypenosides	Shoot apex, leaf	scRNA-seq	Localized saponin pathway genes mainly to mesophyll cells	[68]
*Limonium bicolor*	Salt gland biology	Young leaf	scRNA-seq, trajectory, VIGS	Reconstructed salt gland development	[91]
*Cinnamomum camphora*	Volatile terpenoids	Young leaf	scRNA-seq	Generated leaf cell atlas and terpenoid-related candidate programs	[90]
*Dendrobium* spp.	Polysaccharides, pigments, flavonoids	Stem, leaf, flower	scRNA-seq, MSI	Linked development, pigmentation, and medicinal/quality traits	[70]

### 4.7. Root Epidermis, Endodermis, Pericycle, and Storage Parenchyma

Roots are major medicinal organs in *Panax*, *Salvia*, *Dendrobium*, *Cistanche*, and many other species. Single-cell studies show that root metabolism is closely linked to developmental zones and tissue barriers. In *Panax* root tips, scRNA-seq reconstructed early endodermal differentiation and identified epidermis-specific expression of key ginsenoside enzymes (Figure 3D) [60]. IAA29 was shown to regulate endodermal suberization, while MYB2 and MYB78 positively regulate ginsenoside biosynthesis [60]. In *Salvia*, mature cork cells acquire tanshinone biosynthetic capacity [63]. In *Cistanche deserticola*, scRNA-seq identified parasitism-related cell clusters enriched in horizontally transferred genes and phenylethanoid glycoside-related metabolism, suggesting a connection between host nutrient acquisition and medicinal compound formation [66].

Storage parenchyma and succulent cells are also important in medicinal plants that accumulate polysaccharides, phenylethanoid glycosides, or water-soluble secondary metabolites. In these plants, metabolic function may be closely tied to storage, hydration, and host-derived nutrient conversion.

### 4.8. Mesophyll Cells

Mesophyll cells are often associated with photosynthesis-derived carbon and plastidial isoprenoid metabolism. In *Andrographis paniculata*, scRNA-seq showed that *ApCPS2*, a key diterpenoid pathway gene, is preferentially expressed in photosynthetic mesophyll clusters, and light treatment increased andrographolide accumulation [67]. In *Gynostemma pentaphyllum*, gypenoside biosynthetic genes were enriched in mesophyll cells, suggesting that mesophyll cells may supply carbon and isoprenoid precursors for triterpenoid saponins [68]. In *Taxus* leaves, taxane pathway genes have also been linked to mesophyll or photosynthetically active cells [86,87]. These findings suggest that not all medicinal metabolites are produced in rare secretory structures; some are strongly coupled to photosynthetic tissues.

### 4.9. Floral, Petal, and Reproductive Cells

Flowers and reproductive tissues produce pigments, scent compounds, alkaloids, and other specialized metabolites. In *Catharanthus*, single-cell metabolomics revealed petal cells enriched in flavonoids, anthocyanins, secologanin, or MIAs [45]. In *Dendrobium devonianum*, scRNA-seq and MALDI-MSI connected labellum pigmentation with carotenoid and anthocyanin distribution and identified epidermal trajectories linked to fringe formation [70]. Such studies expand medicinal plant single-cell research beyond classical active ingredients toward quality traits, reproductive ecology, and ornamental value (Figure 3B).

### 4.10. Salt Glands and Specialized Epidermal Accessory Cells

Although salt glands are not classic medicinal metabolite-producing cells, the *Limonium bicolor* salt gland study provides an important model for specialized epidermal cell differentiation (Figure 3E, Table 2). scRNA-seq reconstructed salt gland developmental trajectories and identified cytokinin-related regulation and LbTRY-associated modules controlling salt gland formation [91]. This is relevant to medicinal plants because many biosynthetic structures, including glandular trichomes, secretory epidermal cells, and salt glands, arise from epidermal developmental programs.

**Table 2:** Specialized cell groups in medicinal plant single-cell studies.

Specialized Cell Group	Representative Plants	Metabolites or Biological Focus	Mechanistic Significance	Ref.
Idioblasts	*Catharanthus roseus*	MIAs	Late-pathway activity, alkaloid accumulation, cell-specific regulation	[33,47,48]
Laticifers	*Catharanthus roseus* and latex-producing plants	Alkaloids, latex metabolites	Storage, sequestration, defense-related accumulation	[33,47,48]
Glandular trichomes	*Artemisia annua*, *A. argyi*, *Nepeta tenuifolia*, *Pogostemon cablin*	Artemisinin, essential oils, terpenoids	Secretory biosynthesis and metabolite release	[61,62,69,75]
IPAP/phloem-associated parenchyma	*Catharanthus roseus*	Iridoid intermediates	Upstream pathway compartment and transport origin	[44]
Secretory epidermal cells	*Catharanthus*, *Ophiorrhiza*, *Camptotheca*	MIAs and camptothecin	Biosynthesis, export, and environmental interface	[44,64,88]
Cambium/cork/periderm cells	*Salvia*, *Taxus*, *Cinnamomum*	Tanshinones, taxanes, volatile metabolites	Developmentally acquired metabolic competence	[63,86,90]
Root epidermis and endodermis	*Panax*, *Salvia*	Ginsenosides, phenolics, diterpenoids	Root niche specialization and transport control	[59,60,63]
Mesophyll cells	*Andrographis*, *Gynostemma*, *Taxus*	Diterpenoids, saponins, taxanes	Photosynthesis-coupled precursor supply	[67,68,86]
Storage parenchyma and succulent cells	*Cistanche*	Phenylethanoid glycosides, polysaccharides	Storage and host-derived nutrient transformation	[66]
Floral secretory and pigment cells	*Catharanthus*, *Dendrobium*	Flavonoids, anthocyanins, alkaloids	Reproductive tissue metabolism and quality traits	[48,70]
Salt glands	*Limonium bicolor*	Salt secretion and stress-related programs	Model for specialized epidermal differentiation	[91]
Rare biosynthetic cells	*Hypericum*, *Camptotheca*	Hyperforin, camptothecin intermediates	Missing gene discovery and pathway completion	[58,88]

## 5. Mechanistic Insights from Single-Cell Omics in Medicinal Plants

### 5.1. Multicellular Compartmentalization of Specialized Metabolism

One major mechanistic insight emerging from single-cell omics is that medicinal plant metabolism is frequently organized through the coordinated activity of multiple cell types rather than through uniform pathway expression within a whole organ [25]. This concept was proposed before the single-cell era through anatomical, RNA localization, and enzyme localization studies, but single-cell transcriptomics and single-cell metabolomics now allow pathway modules, candidate transport steps, and metabolite accumulation patterns to be compared across many individual cells [25,44,48].

The monoterpene indole alkaloid pathway in *Catharanthus roseus* remains the best-resolved example of this organization, because early MEP and iridoid genes are associated with internal phloem-associated parenchyma, secologanin and alkaloid scaffold-forming genes are associated mainly with epidermal cells, and late vindoline-related genes and several alkaloid products are associated with idioblasts and laticifers [47,48]. This cellular arrangement indicates that pathway continuity does not necessarily require all enzymes to be co-expressed in one cell type, but instead may depend on the movement of intermediates between metabolically connected cell populations [33,44,47]. A similar principle is emerging from medicinal plants with different chemical classes and organ structures, although the spatial models vary among species [59]. In *Panax*, single-cell and spatial metabolomic studies indicate that ginsenoside-related genes and metabolites are distributed across epidermal, palisade mesophyll, companion cell, phloem parenchyma, root epidermal, and cork-like regions, suggesting a cell-distributed model for triterpenoid saponin metabolism [59,60]. In *Salvia miltiorrhiza*, cross-stage single-cell transcriptomics and AP-MALDI-MSI point to a more tissue-layer-associated model, in which tanshinone biosynthesis and accumulation become strongly linked to mature cork cells during root periderm development [63]. In *Andrographis paniculata*, DESI-MSI and scRNA-seq suggest a photosynthetic-tissue-associated model, where andrographolide-type diterpenoids are enriched in non-veinal leaf regions and outer stem tissues, while *ApCPS2* expression is concentrated in mesophyll-like photosynthetic cell clusters [67]. In *Artemisia annua* and *Artemisia argyi*, glandular trichome-associated cells represent a secretory-structure model, where terpene biosynthesis is linked to specialized epidermal structures rather than broadly distributed leaf cell populations [61,75].

At the single-cell level, pathway continuity is coordinated by complementary source, intermediate-processing, transport, and sink states. Co-expression identifies which cells are competent for a pathway step, whereas transporter expression and subcellular localization nominate interfaces through which precursors or products may move. At the spatial level, adjacency among pathway-active cells, vascular connections, and metabolite gradients constrain plausible transport routes. Developmental synchronization is also important because the abundance and competence of source and storage cells change with organ maturation. A robust model of metabolic coordination therefore requires matched cell-state maps, spatial localization, and metabolite measurements, followed by perturbation or transport assays to distinguish correlation from flux.

These examples suggest that medicinal plant pathways can be organized into several recurring spatial strategies: sequential multi-cell-type partitioning, secretory-cell concentration, tissue-layer enrichment, photosynthesis-associated production, and storage-cell accumulation. Such strategies are not mutually exclusive, because the same pathway may involve biosynthetic cells, transport interfaces, and storage sites that are spatially close but transcriptionally distinct [46,59]. The practical implication is that pathway engineering or breeding cannot rely only on identifying enzyme-coding genes; it also requires knowledge of the cell type in which a gene functions and the cellular route by which intermediates and products move [75]. For example, increasing expression of a pathway enzyme may have limited value if the required precursor is produced in another cell type or if the final compound must be transported to a storage compartment [43,45]. Therefore, single-cell omics reframes medicinal metabolism as a tissue-level coordination problem in which biosynthetic capacity, transport, storage, and regulation are distributed across specialized cellular contexts.

The evidence base remains uneven across taxa and chemical classes. Alkaloid systems, especially Catharanthus, currently provide the strongest support for sequential multicellular partitioning, whereas terpenoid systems more often highlight glandular, mesophyll, cork, or cambial niches. Evidence for saponins, phenylethanoid glycosides, polysaccharides, and volatile mixtures is expanding but remains less mechanistically resolved. The recurring principle is therefore not a universal cell type, but the separation of biosynthesis, transport, and storage across anatomically and developmentally defined cell states. Comparative claims should remain provisional until matched spatial and functional data are available across more plant systems.

### 5.2. Rare Biosynthetic Cells and Pathway Completion

**Figure 4:**
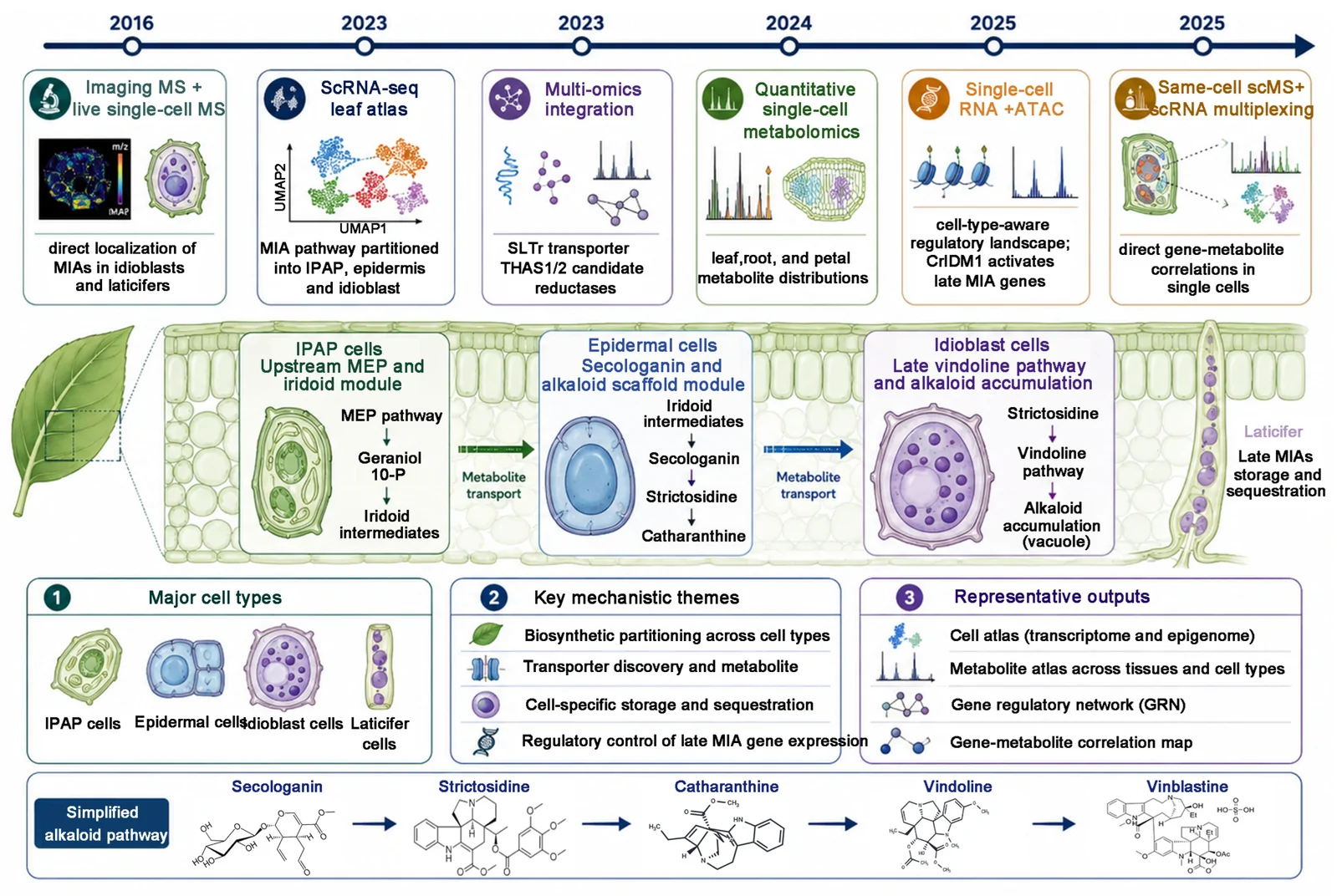
Single-cell research progress in *Catharanthus roseus*. The timeline summarizes advances from imaging and live single-cell mass spectrometry to scRNA-seq, multi-omics integration, quantitative single-cell metabolomics, RNA–ATAC profiling, and same-cell transcriptome–metabolome analysis. Together, these studies resolved the internal phloem-associated parenchyma (IPAP)–epidermis–idioblast–laticifer organization of monoterpene indole alkaloid biosynthesis, identified transport and regulatory mechanisms, and generated cell, metabolite, gene regulatory, and gene–metabolite maps for the pathway.

Many specialized metabolites are produced or accumulated in cell populations that occupy only a small fraction of an organ, and such populations can be difficult to resolve using whole-tissue transcriptomes. Single-cell omics helps address this issue by separating abundant structural or photosynthetic cells from less abundant biosynthetic, secretory, or storage cells, allowing rare metabolic states to be connected with candidate pathway genes [48]. In *Catharanthus roseus*, idioblasts represent a low-abundance but metabolically important cell type because they express cell type, because they express late MIA genes and accumulate several alkaloids that are pharmacologically relevant or biosynthetically related to vinblastine precursors (Figure 4) [25,43,44,47].

**Figure 5:**
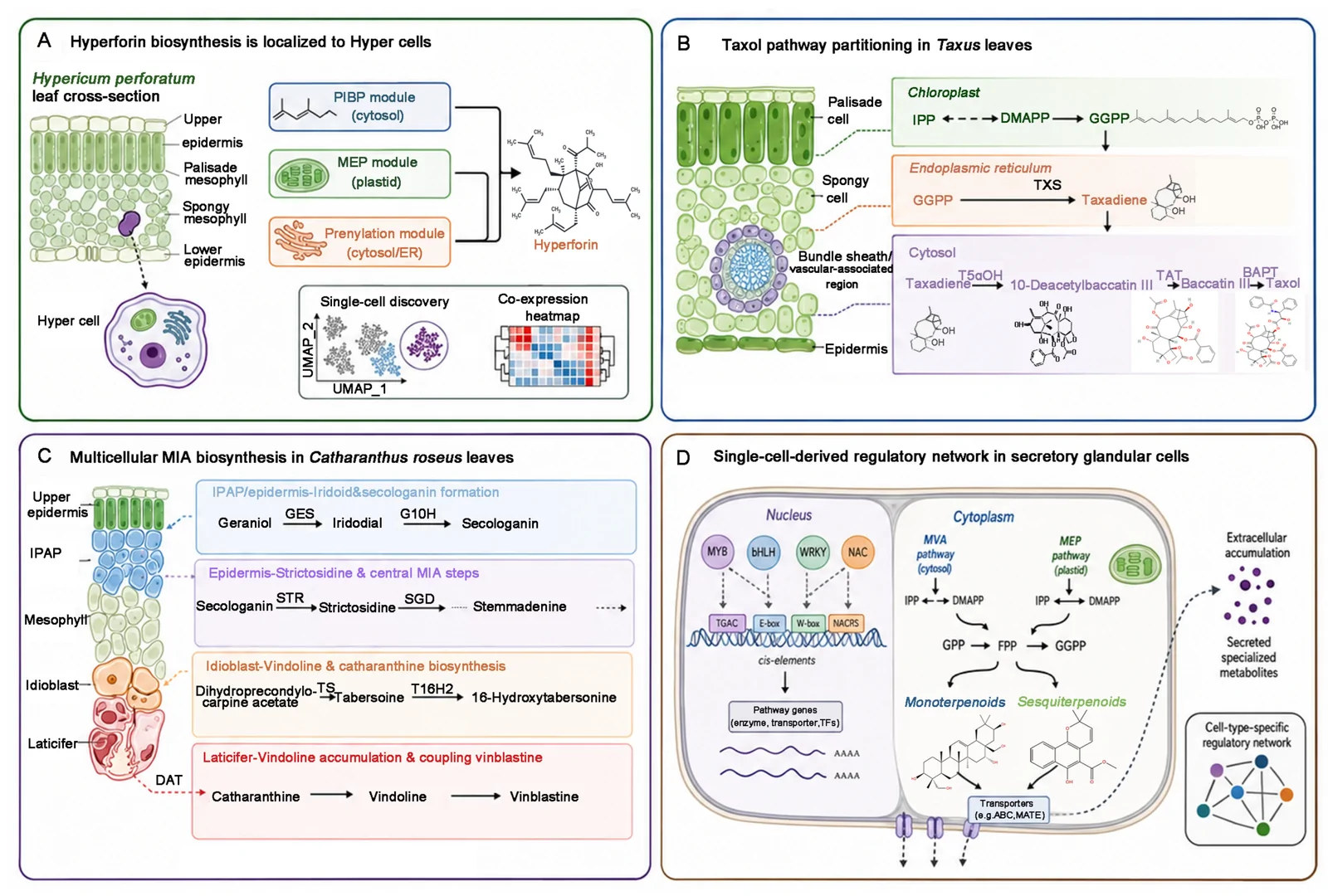
Representative mechanistic insights from single-cell omics in medicinal plants. Single-cell studies reveal rare biosynthetic cells, multicellular pathway partitioning, organelle-specific pathway steps, and cell-type-specific regulatory networks. (**A**). Hyperforin biosynthesis is associated with Hyper cells. (**B**). Taxol formation is distributed across leaf cell types and subcellular compartments. (**C**). Monoterpene indole alkaloid biosynthesis is partitioned among IPAP, epidermal, idioblast, and laticifer cells. (**D**). Secretory glandular cells integrate transcriptional regulation, terpenoid biosynthesis, transport, and extracellular accumulation.

ScRNA-seq and multiome data have made it possible to define these idioblast-associated programs more precisely and to link them with transcriptional regulators and chromatin states [44,48]. The value of rare-cell resolution is especially evident when pathway genes do not show strong co-expression in bulk datasets [58,88]. In *Hypericum perforatum*, scRNA-seq identified Hyper cells that co-express polyketide, MEP, and prenylation modules, and this cellular information supported the identification of prenyltransferases HpPT1–HpPT4 involved in hyperforin biosynthesis (Figure 5A) [58]. This study illustrates how a cell population defined by a pathway module can provide a more informative context for candidate gene discovery than a whole-organ expression profile. A similar logic appears in *Camptotheca acuminata*, where single-nucleus multiome analysis identified STR-positive rare cells with distinct accessible chromatin regions enriched for MYB-binding motifs, suggesting that camptothecin-related biosynthetic activity may be associated with a specialized regulatory state [88]. Although functional validation is still needed for many candidates emerging from rare-cell analyses, such studies show how single-cell resolution can prioritize enzymes, transporters, and transcription factors for biochemical or genetic testing. Rare biosynthetic cells also have broader implications for medicinal plant development and quality improvement. If a small cell population contributes substantially to the formation or storage of a target compound, then variation in the abundance, developmental timing, or activity of that population may influence metabolite yield [44,48,58]. This possibility shifts attention from the average expression of pathway genes in a tissue to the number and state of specific metabolite-producing cells. It also creates opportunities for using cell-type markers, promoter elements, and developmental regulators as tools for selecting or engineering high-producing tissues. For example, the identification of idioblast-associated regulatory programs in *Catharanthus* and Hyper cell programs in *Hypericum* provides a basis for testing whether cell identity and metabolic output can be adjusted together [58]. In this sense, rare-cell analysis does not simply add resolution; it changes the unit of pathway discovery from the organ to the metabolically competent cell state.

### 5.3. Developmental Acquisition of Metabolic Competence

Single-cell trajectory analysis indicates that many medicinal metabolites are linked to cell differentiation rather than to fixed tissue identity alone. A cell may pass through developmental states before acquiring the transcriptional, regulatory, and structural features required for biosynthesis, secretion, or storage [69,75]. This is particularly important for medicinal plants because many active compounds accumulate in organs whose anatomy changes with age, such as roots, periderm, glandular trichomes, flowers, and succulent storage tissues [75].

Trajectory analysis therefore provides a way to connect metabolite formation with developmental timing, rather than treating pathway expression as a static property of an organ [64,69,75]. Glandular trichomes provide a clear example of developmental acquisition of metabolic competence [61,75]. In *Artemisia annua*, single-nucleus and spatial transcriptomic analyses resolved glandular secretory trichome development and showed that artemisinin-related biosynthetic programs are associated with defined secretory cell states within the glandular trichome structure [61]. In *Artemisia argyi*, scRNA-seq of leaf epidermal populations reconstructed a trajectory from epidermal cells toward a glandular trichome-associated state, accompanied by increased expression of lipid metabolism genes, secretory programs, and terpene synthase candidates [75]. These findings suggest that terpene biosynthesis in aromatic medicinal plants is closely linked to the formation and maturation of epidermal secretory structures.

A similar connection between development and metabolism is seen in *Nepeta tenuifolia*, where scRNA-seq identified peltate glandular trichome cells and inferred differentiation trajectories associated with monoterpene biosynthesis [62].

Root and periderm development provide another form of developmental control. In *Salvia miltiorrhiza*, cross-stage single-cell analysis connected pericycle-like cells, phellogen, and mature cork cells, while spatial metabolomics showed that tanshinones accumulate mainly in mature cork layers of older roots [63]. This suggests that tanshinone biosynthesis is linked not only to root identity but also to the progression of secondary growth and cork cell maturation. In *Panax* root tips, integrated single-cell transcriptomics and spatial metabolomics associated ginsenoside biosynthesis with epidermal differentiation and early endodermal development, and functional analysis implicated IAA29, MYB2, and MYB78 in root development and ginsenoside-related regulation [60]. In *Ophiorrhiza pumila*, young epidermal states show stronger expression of camptothecin biosynthetic genes than mature epidermal states, indicating that developmental stage within a cell lineage can influence pathway activity [64]. In *Pogostemon cablin*, scRNA-seq of somatic embryos connected PcNAC048 with lateral root morphogenesis and patchouli alcohol biosynthesis, showing that developmental regulators can also influence specialized metabolism [69]. These studies suggest that medicinal metabolite production is often controlled by developmental competence, which includes cell lineage, maturation stage, tissue age, and differentiation-associated regulatory programs. This view has direct implications for medicinal plant utilization because harvest time, organ age, cultivation condition, and developmental stage can influence the abundance of metabolically competent cells. It also indicates that engineering strategies may need to target developmental regulators, cell-type-specific promoters, or differentiation-associated transcription factors in addition to pathway enzymes. Single-cell trajectory analysis is therefore useful not only for describing cell differentiation, but also for identifying the developmental windows in which medicinal pathways are activated [69,75].

### 5.4. Metabolite Transport and Subcellular Compartmentation

The separation of biosynthetic gene expression and metabolite accumulation is a recurring feature in medicinal plant metabolism, making transport and subcellular compartmentation central mechanistic questions. Transport can occur between organs, tissues, neighboring cell types, or intracellular compartments such as vacuoles, plastids, endoplasmic reticulum, and apoplastic spaces. Because transporters are often members of large gene families and may not show simple expression–metabolite correlations at the organ level, single-cell data provide useful context for prioritizing transporter candidates [64,92].

In medicinal plants, the most relevant transporter families include NPF/NRT1/PTR, ABC, MATE, PUP, amino acid transporters, and other membrane protein families that may participate in precursor import, intermediate exchange, product export, or vacuolar storage. In *Catharanthus roseus*, several transport processes are required to connect the IPAP–epidermis–idioblast pathway architecture [92]. Iridoid intermediates produced in IPAP cells must reach epidermal cells, secologanin must enter the correct intracellular compartment for strictosidine formation, catharanthine appears to move from epidermal biosynthetic contexts to idioblast or surface-associated storage sites, and late alkaloids can accumulate in specialized cells [25,47,92]. Previous functional studies identified transporters such as CrNPF2.9 and ABC-type transporters involved in MIA-related transport, while single-cell and multi-omics analyses provided additional candidates such as SLTr and cell-type-associated transporter genes (Figure 5C) [32,33,92]. The candidate secologanin transporter SLTr was identified by integrating gene clustering, Hi-C, scRNA-seq, and VIGS evidence, illustrating how genomic organization and cell-type information can guide transporter discovery [47]. Subcellular precursor allocation can also influence medicinal pathway flux [64]. In *Ophiorrhiza pumila*, OpAVT1 is located within the STR-TDC gene cluster and encodes a tonoplast amino acid transporter that affects vacuole-to-cytosol tryptophan movement. CRISPR-mediated disruption of OpAVT1 leads to tryptophan accumulation and reduced tryptamine, strictosidine, camptothecin, and related MIA levels, indicating that intracellular amino acid distribution can influence alkaloid biosynthesis [64].

This finding is important because it shows that a pathway-associated gene cluster can include not only enzymes but also transport functions that adjust precursor availability. In *Panax*, candidate ABC and NPF transporters have been discussed in relation to ginsenoside distribution across cell types and tissue regions, although direct biochemical validation of many candidates remains a future task [59,60]. Transporter discovery is likely to become a major area of medicinal plant single-cell research. When metabolites are found in cells that do not strongly express their biosynthetic genes, transporter candidates can be prioritized by comparing source-cell expression, storage-cell expression, metabolite distribution, and subcellular localization. Spatial metabolomics can further reveal metabolite gradients across tissue layers, while scRNA-seq can identify membrane protein genes enriched in candidate transport interfaces. Combining these data with heterologous transport assays, CRISPR, VIGS, and isotope tracing will help distinguish transporters involved in precursor movement, product sequestration, and secretion. Such knowledge is valuable for pathway reconstruction because efficient biosynthesis may require engineering not only enzymes but also the cellular routes that connect substrates, intermediates, and storage compartments [47,64,92].

### 5.5. Cell-Type-Specific Transcriptional Regulation

Cell-type-specific transcriptional regulation provides a mechanistic link between cell identity and medicinal metabolite production. Many well-characterized specialized metabolic pathways are responsive to developmental cues, jasmonate, light, stress, or environmental signals, and these responses are mediated by transcription factor families such as AP2/ERF, bHLH, MYB, WRKY, NAC, HD-ZIP, and bZIP (Figure 5D). Single-cell transcriptomics refines this view by identifying which transcription factors are expressed in the same cell states as biosynthetic genes, while multiome analysis can connect transcription factor expression with accessible regulatory sequences [80,88]. This is important for medicinal plant research because transcription factors may regulate both pathway genes and the differentiation of the cells in which those pathways are active.

The *Catharanthus roseus* MIA pathway illustrates how established regulatory models and newer single-cell models can be combined [48,76]. Jasmonate-responsive transcription factors such as ORCA and BIS regulate parts of the MIA pathway and have been characterized through promoter analysis, overexpression, and functional assays [76]. Single-cell RNA-ATAC analysis added cell-type resolution by identifying idioblast-associated accessible chromatin regions and CrIDM1, a MYB transcription factor that activates late MIA genes such as *D4H* and *DAT* [48]. This indicates that pathway induction and cell-type specificity can involve partially distinct regulatory modules, with some regulators acting broadly in response to signals and others contributing to cell-state-specific expression. Several medicinal plant studies show that developmental regulators can also influence metabolite biosynthesis.

In *Panax* root tips, IAA29 was linked to endodermal suberization, while MYB2 and MYB78 were associated with ginsenoside biosynthesis, connecting root development with triterpenoid saponin metabolism [60]. In *Pogostemon cablin*, PcNAC048 was identified through scRNA-seq of somatic embryos and shown to promote lateral root formation while repressing *PcPTS* promoter activity and patchouli alcohol accumulation [69]. In *Ophiorrhiza pumila*, cell-type-specific expression of camptothecin pathway genes in young epidermal cells provides a basis for identifying transcription factors that regulate epidermal alkaloid biosynthesis [64]. In *Artemisia argyi*, MYB, bHLH, WRKY, and HD-ZIP candidates were associated with glandular trichome development and terpene synthase expression, although genetic validation is still needed for many of these candidates [75]. In cotton secretory glandular cells, GoHSFA4a and GoNAC42 regulate terpenoid metabolism, providing a useful example of how single-cell-based regulatory network analysis can identify transcription factors acting in specialized secretory cells [80].

Cell-type-specific regulation also provides practical targets for metabolic engineering. Promoters active in idioblasts, glandular trichomes, cork cells, epidermal cells, or root storage cells could allow pathway genes to be expressed in metabolically suitable tissues while reducing effects in unrelated cell types. Transcription factors that control both cell differentiation and pathway expression may be useful for increasing the abundance or activity of biosynthetic cells, but they require careful functional analysis because they can also influence growth or organ development. Single-cell omics therefore helps prioritize regulators not only by their expression level, but by their position within cell-type-specific networks and developmental trajectories [60,69,75].

### 5.6. Chromatin Accessibility and Regulatory Landscapes

Single-cell chromatin accessibility provides an additional layer for understanding why medicinal pathway genes are active in some cell types but not others. ATAC-seq identifies open chromatin regions that can include promoters, enhancers, transcription factor binding regions, and other *cis*-regulatory elements, and scATAC-seq assigns these regulatory features to specific cell states.

In specialized plant metabolism, this is particularly relevant because pathway genes often respond to hormones or environmental signals while also being restricted to defined developmental or anatomical contexts. Thus, chromatin accessibility can help distinguish whether cell-type-specific pathway expression is associated with local promoter accessibility, motif enrichment, or broader regulatory states [48,49].

In *Catharanthus roseus*, single-cell RNA-ATAC multiome analysis showed that idioblast-associated chromatin regions are enriched for MYB and WRKY motifs and are linked to late MIA gene expression [48]. This regulatory information supported the identification of CrIDM1 as an idioblast-associated MYB transcription factor that activates *D4H* and *DAT*, two late vindoline pathway genes. The study also showed that RNA-based clustering and ATAC-based clustering can provide complementary views of cell identity, because accessible chromatin may be shared among related cells while transcript abundance more directly reflects active metabolic programs.

In *Camptotheca acuminata*, single-nucleus multiome analysis identified STR-positive cells with distinct accessible chromatin regions, and motif analysis suggested a role for MYB-related regulation in rare camptothecin-associated cells [88]. These studies indicate that rare biosynthetic cells can be defined not only by marker gene expression but also by regulatory landscapes that may control pathway activation. Chromatin-based information has several uses for medicinal plant development. First, accessible regions near pathway genes can help to identify candidate promoters or enhancer-like sequences suitable for cell-type-selective expression [88]. Second, motif enrichment can prioritize transcription factor families for functional testing, especially when scRNA-seq alone produces many candidate regulators. Third, integration of chromatin accessibility with gene clusters and 3D genome organization can reveal whether pathway genes are regulated as local genomic units or as dispersed modules coordinated by shared transcription factors [48].

In *Catharanthus*, the integration of high-quality genome assembly, Hi-C, scRNA-seq, and scMS helped to connect MIA gene organization with candidate transport and biosynthetic functions. This suggests that future medicinal plant studies may benefit from combining scATAC, chromatin interaction data, and metabolite localization to understand how regulatory architecture contributes to pathway activity. A key future direction is to move from motif enrichment to regulatory validation. Candidate transcription factors and *cis*-regulatory regions identified by scATAC should be tested using reporter assays, transient expression, genome editing, DAP-seq, yeast one-hybrid assays, or cell-type-specific expression systems [48]. This will be important because motif enrichment alone cannot determine whether a transcription factor directly activates a pathway gene in the relevant cell type. When combined with functional testing, single-cell chromatin accessibility can provide a means of identifying regulatory parts for synthetic biology and breeding, including cell-type-specific promoters and transcription factors that guide pathway expression to appropriate cellular contexts.

### 5.7. Single-Cell Metabolomics Reveals Divergence Between Biosynthesis and Storage

Single-cell metabolomics directly addresses a question that transcriptomics alone cannot resolve: whether a metabolite accumulates in the same cell type that expresses its biosynthetic genes. This distinction is central to medicinal plants because active compounds can be synthesized, modified, transported, and stored in different cells or subcellular compartments. If transcript and metabolite patterns match, this supports local biosynthesis and accumulation; if they differ, this can indicate transport, conversion, storage, secretion, or developmental timing effects. Single-cell metabolomics therefore provides an important bridge between pathway gene discovery and the chemical phenotype that defines medicinal quality.

In *Catharanthus roseus*, single-cell metabolomics has provided several examples of gene–metabolite agreement and divergence [25,45]. Imaging MS and live single-cell MS showed that several alkaloids, including catharanthine, ajmalicine, serpentine, and strictosidine-related compounds, are enriched in idioblasts and laticifers, whereas secologanin is enriched in epidermal cells [25]. Later single-cell metabolomics indicated that alkaloid localization is developmentally and spatially complex, supporting the idea that MIA distribution changes with tissue context and cell state [43]. Quantitative plate-based single-cell mass spectrometry measured 16 natural products across leaf, root, and petal cells and showed that metabolite concentrations and co-accumulation patterns can vary strongly among individual cells [45]. Same-cell scMS and scRNA-seq further allowed direct correlation of metabolite levels with gene expression in individual cells, showing that vindoline and anhydrovinblastine correlate with idioblast-enriched late pathway genes, whereas catharanthine accumulation is less directly correlated with its epidermal biosynthetic genes [46]. This pattern is consistent with a model in which some metabolites are synthesized and stored locally, while others are transported after synthesis.

Spatial metabolomics extends this logic from isolated cells to tissue regions. In *Salvia miltiorrhiza*, AP-MALDI-MSI showed that tanshinones are enriched in the cork layer of mature roots, complementing scRNA-seq evidence for cork-associated diterpenoid biosynthesis [63]. In *Panax*, MALDI-MSI and MALDI-2-MSI mapped ginsenosides and related compounds across root, stem, leaf, epidermal, cork-like, and vascular-associated regions, supporting the view that ginsenoside metabolism involves spatially distributed biosynthetic and storage contexts [8]. In *Taxus*, integrated MSI and single-cell transcriptomics provided spatial evidence for tissue-specific taxane biosynthesis and transport in leaves and stems [86]. In *Andrographis paniculata*, DESI-MSI mapped andrographolide-type diterpenoids to non-veinal leaf regions and outer stem tissues, while scRNA-seq associated a key diterpenoid gene with mesophyll cell clusters [67]. For medicinal plant development, these results have two implications. First, metabolite accumulation should not be used as a direct proxy for biosynthetic gene expression without considering transport and storage. Second, improving medicinal compound yield may require modifying the capacity of storage cells or transport interfaces, not only increasing pathway enzyme expression. The integration of single-cell metabolomics, scRNA-seq, spatial metabolomics, and functional assays can therefore define whether a target compound is limited by enzyme activity, precursor supply, transport, storage capacity, or developmental timing. As more medicinal plants adopt same-cell transcriptome–metabolome profiling, it should become possible to build gene–metabolite maps that distinguish biosynthetic cells, storage cells, and transport-connected cell states with greater precision.

Spatial separation between biosynthesis and storage can provide several physiological advantages. It can protect biosynthetic cells from autotoxic or highly reactive products, reduce feedback inhibition of pathway enzymes, and concentrate compounds in vacuoles, epidermal surfaces, latex, or secretory cavities where they can function in defense, wound sealing, or interactions with herbivores and microbes. Separation also allows plants to deploy metabolites at anatomical interfaces while maintaining precursor generation in photosynthetic or vascular-associated source cells. The observed mismatch is therefore not merely a technical discrepancy between omics layers; it can reflect an evolved division of labor that couples chemical defense and storage capacity to tissue function. These interpretations remain hypotheses unless intercellular flux is demonstrated by transporter perturbation, isotope tracing, or matched spatial measurements.

## 6. Future Perspectives

### 6.1. Building Comparative Cell Atlases Around Medicinal Organs, Chemistry, and Development

The rapid growth of medicinal plant single-cell studies now makes it possible to move from isolated species-specific atlases toward comparative cell atlases organized around medicinal organs, chemical classes, and developmental stages.

A practical direction is not simply to sequence more species, but to select species that represent major medicinal organs and pathway types, such as alkaloid-producing leaves and shoots, terpenoid-producing trichomes and bark tissues, saponin-rich roots, polysaccharide-rich storage organs, and volatile-oil-producing aerial tissues. For alkaloid-producing plants, comparative analysis across *Catharanthus roseus*, *Ophiorrhiza pumila*, *Camptotheca acuminata*, and related systems could clarify how epidermal cells, idioblasts, vascular-associated cells, and rare STR-positive cells contribute to alkaloid biosynthesis, storage, and transport [44,47,48].

For terpenoid-producing plants, studies across *Taxus*, *Artemisia*, *Salvia*, *Andrographis*, *Pogostemon*, and *Cinnamomum* could test how glandular trichomes, cork cells, mesophyll cells, cambium-associated cells, and epidermal or secretory tissues support different terpenoid pathways [55]. For saponin- and storage-metabolite-rich plants, *Panax*, *Gynostemma*, *Dendrobium*, and *Cistanche* provide useful systems for connecting root, stem, storage parenchyma, epidermal, and vascular cell states with compound accumulation. These atlases should include not only the harvested medicinal organs, but also developmental stages before and during metabolite accumulation, because several studies indicate that biosynthetic activity can be associated with specific differentiation states rather than with organ identity alone. A feasible experimental design would compare high-accumulation and low-accumulation tissues across a developmental series, while using matched metabolite quantification to define when and where the target compound begins to accumulate. Such comparative atlases would provide cell markers, pathway-active states, candidate promoters, and developmental indicators that can be tested in breeding, cultivation, and metabolic engineering studies.

### 6.2. Technical Challenges and Practical Solutions for Metabolite-Rich Tissues

The usefulness of a medicinal plant cell atlas depends on whether the relevant biosynthetic, transport, and storage cells are recovered with sufficient quality. Protoplast-based scRNA-seq has been effective in many young, soft plant tissues, but medicinal organs often include lignified cells, suberized layers, wax-rich epidermis, mucilage, latex, resin, essential oils, phenolics, and abundant specialized metabolites that can influence digestion efficiency and cell viability. Single-nucleus RNA-seq provides a practical alternative for woody tissues, mature leaves, roots, bark-like organs, needles, trichome-containing tissues, and frozen samples, as shown by recent studies in *Taxus*, *Panax*, *Artemisia annua*, and *Camptotheca*. However, nuclei-based data should be interpreted together with transcriptomic and spatial evidence when possible, because nuclear RNA and cytoplasmic mRNA can differ in abundance for some genes [42]. Future studies should therefore treat protoplast-based and nuclei-based methods as complementary options, rather than choosing one method as a universal solution.

A practical workflow for difficult medicinal tissues would combine parallel optimization of protoplast isolation, nuclei isolation, histological observation, and spatial metabolite mapping. For each species, recovery of expected specialized cells should be checked using microscopy, marker genes, anatomical features, and metabolite localization. For example, laticifers in *Catharanthus roseus* were detected by single-cell metabolomics and imaging MS, but they are not always well represented in droplet-based protoplast transcriptomes, indicating that cell recovery should be evaluated against known tissue anatomy. For glandular trichomes, enrichment of trichome tissues, nuclei isolation, and spatial transcriptomic validation may provide more reliable access to secretory cells than whole-leaf protoplasting alone. For cork, cambium, bark, resin-associated tissues, and storage organs, single-nucleus sequencing combined with MSI or targeted dissection may better preserve the relationship between tissue organization and metabolite distribution. Establishing tissue-specific sample preparation guidelines would improve the comparability of medicinal plant single-cell datasets and reduce the risk of overlooking metabolically important cells because of technical under-recovery.

Method choice should be guided by a pilot recovery benchmark rather than by tissue convenience alone. Parallel protoplast and nucleus preparations can be compared for viability, ambient RNA, doublet rate, detected genes, representation of anatomy-defined cell classes, and recovery of known specialized-cell markers. When either preparation under-recovers a key lineage, enrichment, laser microdissection, targeted spatial profiling, or MSI can provide complementary evidence. Reporting these benchmarks would make technical bias visible and improve reproducibility across species.

### 6.3. Integrating Transcriptomics, Chromatin Accessibility, Metabolomics, and Spatial Information

**Figure 6:**
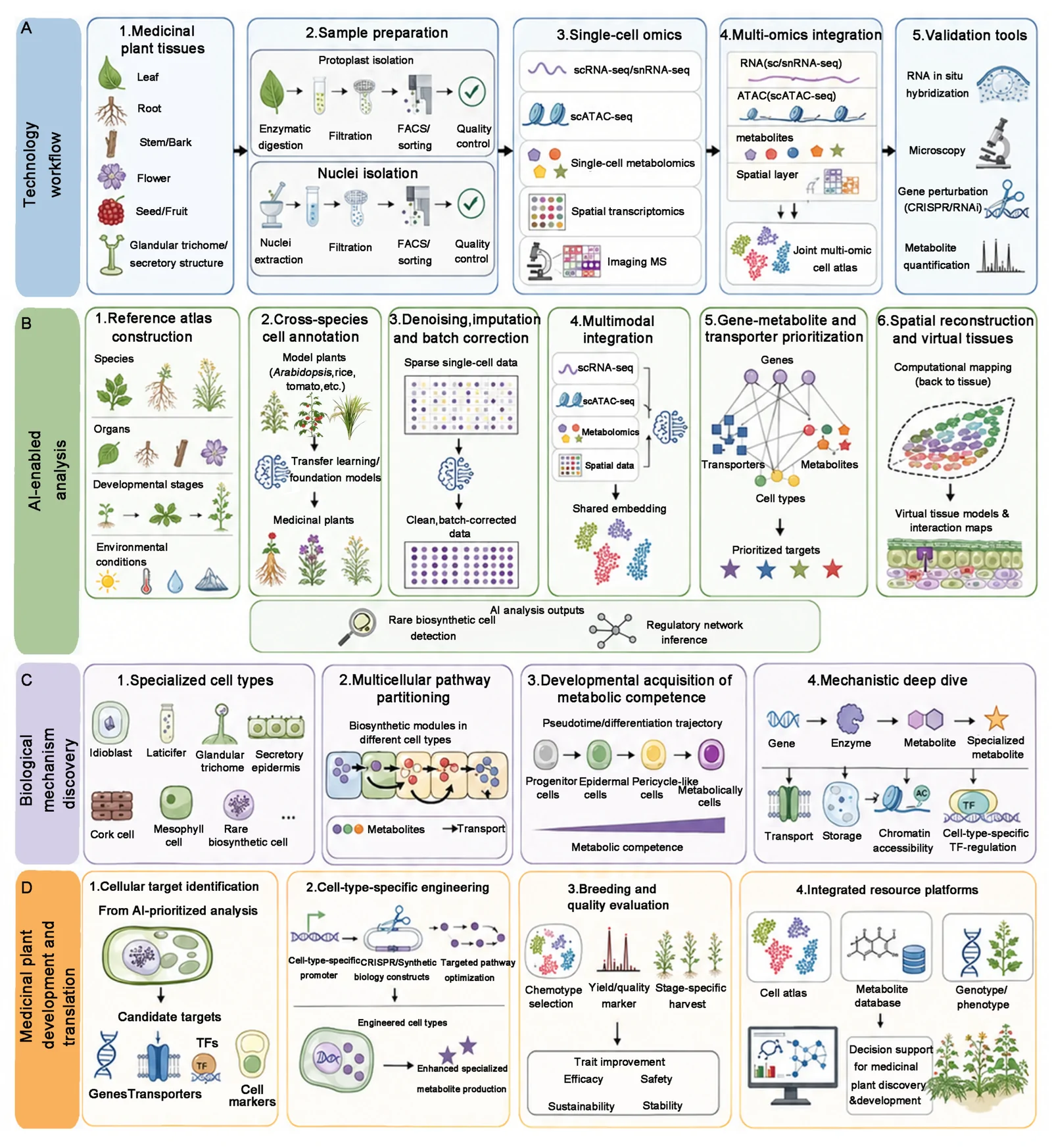
Single-cell omics and AI for medicinal plant research and development. (**A**). Technology workflow. Medicinal plant tissues are processed through protoplast or nuclei isolation, followed by single-cell or single-nucleus transcriptomics, scATAC-seq, single-cell metabolomics, spatial transcriptomics, imaging mass spectrometry, multi-omic integration, and experimental validation. (**B**). AI-enabled analysis. Artificial intelligence supports reference atlas construction, cross-species cell annotation, denoising, batch correction, multimodal integration, gene–metabolite association, transporter prioritization, regulatory network inference, and spatial reconstruction. (**C**). Biological mechanism discovery. Integrated single-cell data reveal specialized cell types, multicellular pathway partitioning, developmental acquisition of metabolic competence, transport, storage, chromatin accessibility, and cell-type-specific transcriptional regulation. (**D**). Medicinal plant development and translation. Cell-resolved discoveries guide target identification, cell-type-specific engineering, breeding, quality evaluation, stage-specific harvesting, and integrated resource platforms for natural product discovery and sustainable production.

Future medicinal plant studies should increasingly use integrated designs because biosynthetic gene expression, metabolite accumulation, and anatomical storage sites are often not identical (Figure 6). scRNA-seq and snRNA-seq define cell populations and pathway gene expression, but they cannot by themselves determine whether a metabolite accumulates in the same cell type. Single-cell metabolomics and MSI provide chemical localization, but their mechanistic interpretation is strengthened when they are combined with cell-type-resolved transcriptomes and tissue anatomy. ScATAC-seq and scRNA multiome profiling add a regulatory layer by connecting pathway-active cell states with accessible chromatin regions, promoter features, and candidate transcription factor motifs. A feasible multi-omics design would collect matched tissues from the same organ, developmental stage, and treatment for scRNA-seq or snRNA-seq, targeted metabolite quantification, spatial metabolomics, and histological annotation. When a cell population shows strong pathway activity or rare biosynthetic signatures, scATAC-seq or RNA–ATAC multiome profiling can be added to identify candidate regulatory regions and transcription factors. In *Catharanthus roseus*, the integration of scRNA-seq, single-cell metabolomics, chromatin interaction, single-cell multiome analysis, and same-cell transcriptome–metabolome profiling has connected pathway partitioning, metabolite storage, transport candidates, and regulatory programs.

In *Panax* and *Salvia*, the combination of single-cell transcriptomics and spatial metabolomics has clarified how ginsenosides and tanshinones relate to root epidermal regions, cork-like tissues, vascular-associated regions, and periderm development. In *Andrographis* and *Taxus*, pairing MSI with single-cell or single-nucleus expression data has provided spatial context for diterpenoid and taxoid biosynthesis. The next step is to improve experimental matching across modalities, because datasets generated from different organs or stages are valuable but can be difficult to integrate into a specific mechanistic model. Same-cell transcriptome–metabolome profiling, currently demonstrated in *Catharanthus*, should be extended to additional chemical classes when metabolite sensitivity, standards, and cell recovery are suitable.

### 6.4. Connecting Cell-Resolved Candidates to Functional Validation

Single-cell omics can prioritize biosynthetic genes, transcription factors, transporters, and cell markers, but these candidates need functional testing before they can be used confidently in medicinal plant development [76]. For enzymes, rapid validation can be performed through transient expression in *Nicotiana benthamiana*, yeast or microbial reconstruction, purified enzyme assays, or hairy root systems. For transporters, heterologous transport assays, subcellular localization, gene disruption, metabolite profiling, and isotope feeding can help distinguish precursor import, intermediate transport, product export, and vacuolar storage. For transcription factors, promoter activation assays, yeast one-hybrid assays, DAP-seq, VIGS, CRISPR-based perturbation, overexpression, and cell-type-specific reporter constructs can test whether candidate regulators directly control pathway genes or influence the development of metabolite-producing cells. Perturbation-informed single-cell analysis is a particularly useful direction because it links candidate genes with cell-state responses. In *Taxus*, multiplexed single-nucleus profiling under pathway perturbation supported the identification of genes required for baccatin III biosynthesis and enabled reconstruction of baccatin III production in *Nicotiana benthamiana*. This strategy could be adapted to additional medicinal plants by applying elicitors, hormones, light treatments, developmental sampling, or targeted genetic perturbations before single-cell profiling. Jasmonate-responsive alkaloid and terpene pathways, light-responsive diterpenoid pathways, and developmentally regulated cork or trichome pathways are suitable contexts for linking perturbation, cell state, and pathway activation. A realistic validation pipeline would begin with cell-resolved candidate ranking, proceed to rapid transient or heterologous assays, and then use VIGS, CRISPR, or stable transformation in the native or related system for selected candidates. This approach would make single-cell omics more directly connected to medicinal plant breeding, cultivation improvement, and synthetic biology (Table 3).

### 6.5. Establishing Data Standards for AI-Ready Medicinal Plant Single-Cell Resources

The increasing number of medicinal plant single-cell datasets creates an opportunity for cross-species integration, but this requires consistent metadata, quality metrics, and annotation practices. Each dataset should report species, accession or cultivar, organ, developmental stage, tissue position, treatment, harvest condition, cell or nuclei isolation method, sequencing chemistry, quality-control thresholds, reference genome version, annotation strategy, marker evidence, and metabolite measurement method. For metabolomics and MSI datasets, metabolite annotation confidence, standards, ion mode, spatial resolution, sample preparation, and tissue orientation should be documented in a way that can be linked to cell-type data. For scATAC-seq and multiome datasets, peak calling, motif analysis, promoter definitions, genome annotation quality, and integration methods should be described consistently. These standards are important because medicinal plants often differ in genome quality, gene naming systems, metabolite nomenclature, cell markers, and available functional evidence. A shared framework for cell-type labels, evidence levels, pathway annotations, and metabolite identifiers would make it easier to build searchable medicinal plant cell atlases. Cell types such as epidermis, mesophyll, phloem parenchyma, xylem, endodermis, pericycle, cambium, idioblasts, glandular trichomes, laticifers, cork cells, storage parenchyma, and secretory cells should be annotated using a combination of conserved markers, species-specific markers, spatial evidence, metabolite evidence, and functional validation where available. Such resources would support both manual interpretation and machine-learning-based annotation, while allowing uncertainty to be retained when a cell type is inferred rather than directly validated. A practical near-term goal would be to create a curated medicinal plant single-cell reference table that links each dataset to species, tissue, method, cell type, pathway, metabolite, and validation evidence.

### 6.6. Applying AI to Annotation, Gene–Metabolite Association, and Candidate Ranking

AI-assisted methods can help analyze medicinal plant single-cell datasets because these datasets span many species, organs, platforms, chemical classes, and annotation systems. For cell-type annotation, transfer learning and probabilistic models can help compare conserved cell identities across species while also highlighting lineage-specific states such as idioblasts, glandular trichomes, Hyper cells, STR-positive cells, cork-associated biosynthetic cells, and storage parenchyma [93]. For cross-species comparison, domain adaptation and spatial alignment methods can reduce technical differences while preserving biologically meaningful variation among medicinal organs and pathway-active cell states [94,95]. These tools should be used with anatomical validation and marker-gene evidence, because AI-based labels are best treated as testable annotations rather than final biological assignments [95,96]. AI can also support gene–metabolite association and candidate prioritization. Multimodal models can integrate gene expression, chromatin accessibility, metabolite abundance, pathway annotations, gene family membership, subcellular localization, and spatial position to rank enzymes, transcription factors, and transporters. Graph-based models are well suited for representing relationships among genes, metabolites, cell types, regulatory elements, and transport routes, although curated medicinal plant training data will be needed for reliable predictions. For transporter prioritization, computational models could combine cell-type enrichment, membrane protein annotation, co-localization with metabolite gradients, pathway topology, and perturbation responses. For metabolite annotation, tools such as SIRIUS and Spec2Vec can support MS/MS-based formula prediction and spectral similarity analysis, which is useful for connecting single-cell or spatial metabolomics to pathway hypotheses [97,98]. Protein structure prediction tools such as AlphaFold and AlphaFold 3 can support hypothesis generation for enzymes and transporters, although biochemical assays remain necessary for functional assignment [99,100]. A realistic near-term use of AI is therefore evidence-based candidate prioritization, not automated pathway discovery.

To date, machine learning in medicinal-plant single-cell studies has been used mainly through established computational components rather than through plant-specific foundation models. Unsupervised clustering and dimensionality reduction have delineated rare biosynthetic populations; trajectory and graph-based analyses have reconstructed epidermal, trichome, endodermal, periderm, and somatic-embryo transitions; and multimodal correlation or candidate-ranking workflows have connected cell states with chromatin accessibility, metabolite abundance, and pathway genes. Examples include idioblast and regulatory-state mapping in Catharanthus, rare STR-positive cells in Camptotheca, glandular-trichome trajectories in Artemisia and Nepeta, root differentiation in Panax, and Hyper-cell-guided pathway discovery in Hypericum [45,49,59,61,62,63,76,84]. These applications are useful precedents, but most remain dataset-specific and should not be described as general-purpose AI.

The main limitation is the scarcity of benchmarked, high-quality gold-standard datasets for non-model medicinal plants. Reference genomes, cell-type labels, metabolite annotations, and experimentally validated gene-metabolite links are uneven, while datasets are often small, tissue-specific, and affected by dissociation or platform bias. Under these conditions, models can produce false-positive gene-metabolite or transporter links because co-expression, spatial co-localization, and correlated responses do not establish biochemical function or transport direction. Credible evaluation should therefore use held-out species or tissues, negative controls, uncertainty estimates, and independent anatomical, biochemical, or genetic validation. AI outputs should be treated as ranked hypotheses, not as pathway assignments.

### 6.7. Developing Spatial AI and Virtual Medicinal Plant Tissues

Spatial AI provides a practical route to connect dissociated single-cell data with tissue organization. Single-cell transcriptomes lose physical position, whereas MSI and spatial transcriptomics retain tissue context but may have lower molecular coverage or lower cell-type resolution. Deconvolution tools can map single-cell-defined cell types onto spatial transcriptomic spots or tissue regions, while image-based segmentation can help define anatomical boundaries for interpreting metabolite gradients. In medicinal plants, such approaches could be used to map pathway-active cells onto cork layers, glandular trichomes, vascular bundles, root tips, stem bark, flower tissues, and storage organs (Table 3).

A realistic spatial AI workflow would combine histological images, MSI maps, scRNA-seq or snRNA-seq clusters, pathway gene scores, and metabolite intensities from serial or adjacent tissue sections. The output would be a virtual tissue map in which each region is annotated with probable cell types, pathway activity, metabolite abundance, and candidate transport interfaces. Such maps could help distinguish whether a target compound is associated with biosynthetic cells, storage cells, vascular transport routes, epidermal secretion, or developmental boundaries. This direction is experimentally feasible because it builds on existing spatial and single-cell datasets rather than requiring a completely new technology platform. Near-term applications include analysis of ginsenoside gradients in *Panax* roots, tanshinone enrichment in *Salvia* cork layers, taxane distribution in *Taxus* stems, and andrographolide distribution in *Andrographis* leaves and stems, and pigment or alkaloid distribution in floral tissues.

### 6.8. From Cell-Resolved Mechanisms to Medicinal Plant Development

The long-term value of medicinal plant single-cell omics will depend on whether cell-resolved mechanisms can be translated into practical development strategies. Several near-term applications are realistic. First, cell-type markers and pathway gene scores can be used to identify tissues, developmental stages, or cultivation conditions associated with a higher abundance of biosynthetically active cells. Second, cell-type-specific promoters or regulatory regions can be used to drive pathway genes in suitable biosynthetic or storage contexts. Third, transporter candidates can be tested to improve precursor supply, product sequestration, or secretion in native plants and heterologous systems. Fourth, developmental regulators can be evaluated for their ability to alter the abundance or activity of metabolite-producing cell types while monitoring growth and organ formation. Fifth, cell-resolved pathway maps can guide synthetic biology by indicating whether a pathway should be reconstructed in one compartment, divided between compartments, or paired with transport and storage modules.

These applications should be considered together with targeted metabolite quantification, functional assays, genotype comparisons, cultivation data, and quality-control standards. Single-cell omics does not replace pharmacognosy, phytochemistry, breeding, or synthetic biology, but it provides cellular context that can help these approaches identify more precise targets and experimental conditions. As medicinal plant cell atlases become better integrated with metabolite data and functional validation, they should support more informed strategies for natural product discovery, medicinal plant improvement, and sustainable production of plant-derived compounds.

## 7. Conclusions

Single-cell and spatial multi-omics are clarifying how metabolites are produced, transported, regulated, and stored across specialized cell types and developmental states. By integrating transcriptomes, chromatin accessibility, metabolite profiles, and spatial context, these approaches reveal pathway compartmentalization, rare biosynthetic cells, regulatory networks, and transport processes that are obscured in bulk analyses. Future progress will depend on comparative cell atlases, improved sampling of specialized tissues, functional validation, standardized datasets, and AI-assisted integration. Together, these advances will support natural-product discovery, medicinal plant improvement, metabolic engineering, and sustainable production of bioactive compounds.

## Figures and Tables

**Table 3:** AI and machine-learning opportunities in medicinal plant single-cell omics.

AI Task	Input Data	Representative Methods	Application	Expected Output
Cell-type annotation	scRNA-seq, snRNA-seq	Transfer learning, foundation models	Annotate rare or lineage-specific cell states	Candidate cell atlas
Cross-species integration	Multi-species cell atlases	Domain adaptation, contrastive learning	Compare medicinal and model plants	Provisional cell programs
Trajectory inference	Developmental single-cell data	Neural ODEs, graph methods	Reconstruct secretory cell development	Lineage hypothesis
Regulatory network inference	scRNA-seq + scATAC-seq	Graph neural networks, Bayesian models	Identify TFs controlling metabolic pathways	Prioritized regulators
Gene-metabolite association	scRNA-seq + scMS	Multimodal fusion, matrix completion	Connect pathway genes with metabolites	Candidate biosynthetic genes
Transporter prediction	Omics + localization + metabolite gradients	Graph learning, candidate ranking	Discover intercellular transporters	Transporter shortlist
Spatial reconstruction	Single-cell + spatial transcriptomics/MSI	Deconvolution, diffusion models	Map metabolite-producing niches	Probabilistic tissue map
Literature mining	Papers, databases, genomes	LLMs, knowledge graphs	Curate pathway evidence	Structured medicinal plant knowledge base
Engineering design	Multi-omics + phenotype data	Generative models, active learning	Prioritize metabolic engineering targets	Engineering hypotheses

## Data Availability

The original contributions presented in this study are included in the article. Further inquiries can be directed to the corresponding author.

## Abbreviations

ABC: ATP-binding cassette; AI: artificial intelligence; AP-MALDI-MSI: atmospheric-pressure matrix-assisted laser desorption/ionization mass spectrometry imaging; CRISPR: clustered regularly interspaced short palindromic repeats; DAP-seq: DNA affinity purification sequencing; DESI-MSI: desorption electrospray ionization mass spectrometry imaging; FACS: fluorescence-activated cell sorting; Hi-C: high-throughput chromosome conformation capture; IPAP: internal phloem-associated parenchyma; MALDI-MSI: matrix-assisted laser desorption/ionization mass spectrometry imaging; MALDI-2-MSI: matrix-assisted laser desorption/ionization-2 mass spectrometry imaging; MEP: methylerythritol phosphate; MIA: monoterpene indole alkaloid; MSI: mass spectrometry imaging; MS/MS: tandem mass spectrometry; NPF: NRT1/PTR FAMILY; PRISMA: Preferred Reporting Items for Systematic Reviews and Meta-Analyses; RNA-seq: RNA sequencing; scATAC-seq: single-cell assay for transposase-accessible chromatin sequencing; scMS: single-cell mass spectrometry; scRNA-seq: single-cell RNA sequencing; STR: Strictosidine Synthase; snRNA-seq: single-nucleus RNA sequencing; VIGS: virus-induced gene silencing; WoS: Web of Science; MATE: multidrug and toxic compound extrusion; PUP: purine uptake permease; NRT1/PTR: nitrate transporter 1/peptide transporter; AP2/ERF: APETALA2/ethylene-responsive factor.

## References

[1] Atanasov A.G., Zotchev S.B., Dirsch V.M., Supuran C.T. (2021). Natural products in drug discovery: Advances and opportunities. Nat. Rev. Drug Discov..

[2] Guo L., Yao H., Chen W., Wang X., Ye P., Xu Z., Zhang S., Wu H. (2022). Natural products of medicinal plants: Biosynthesis and bioengineering in post-genomic era. Hortic. Res..

[3] Weng J.K., Lynch J.H., Matos J.O., Dudareva N. (2021). Adaptive mechanisms of plant specialized metabolism connecting chemistry to function. Nat. Chem. Biol..

[4] Li F.S., Weng J.K. (2017). Demystifying traditional herbal medicine with modern approach. Nat. Plants.

[5] Guo L., Winzer T., Yang X., Li Y., Ning Z., He Z., Teodor R., Lu Y., Bowser T.A., Graham I.A. (2018). The opium poppy genome and morphinan production. Science.

[6] Rai A., Saito K., Yamazaki M. (2017). Integrated omics analysis of specialized metabolism in medicinal plants. Plant J..

[7] Li B., Bhandari D.R., Janfelt C., Römpp A., Spengler B. (2014). Natural products in *Glycyrrhiza glabra* (licorice) rhizome imaged at the cellular level by atmospheric pressure matrix-assisted laser desorption/ionization tandem mass spectrometry imaging. Plant J..

[8] Taira S., Ikeda R., Yokota N., Osaka I., Sakamoto M., Kato M., Sahashi Y. (2010). Mass spectrometric imaging of ginsenosides localization in Panax ginseng root. Am. J. Chin. Med..

[9] O’Connor S.E., Maresh J.J. (2006). Chemistry and biology of monoterpene indole alkaloid biosynthesis. Nat. Prod. Rep..

[10] De Luca V., Salim V., Atsumi S.M., Yu F. (2012). Mining the biodiversity of plants: A revolution in the making. Science.

[11] Croteau R., Ketchum R.E., Long R.M., Kaspera R., Wildung M.R. (2006). Taxol biosynthesis and molecular genetics. Phytochem. Rev..

[12] Lange B.M., Ahkami A. (2013). Metabolic engineering of plant monoterpenes, sesquiterpenes and diterpenes--current status and future opportunities. Plant Biotechnol. J..

[13] Tholl D. (2015). Biosynthesis and biological functions of terpenoids in plants. Adv. Biochem. Eng. Biotechnol..

[14] Saito K., Matsuda F. (2010). Metabolomics for functional genomics, systems biology, and biotechnology. Annu. Rev. Plant Biol..

[15] Fernie A.R., Tohge T. (2017). The Genetics of Plant Metabolism. Annu. Rev. Genet..

[16] Tohge T., Fernie A.R. (2017). Leveraging Natural Variance towards Enhanced Understanding of Phytochemical Sunscreens. Trends Plant Sci..

[17] Schläpfer P., Zhang P., Wang C., Kim T., Banf M., Chae L., Dreher K., Chavali A.K., Nilo-Poyanco R., Bernard T. (2017). Genome-Wide Prediction of Metabolic Enzymes, Pathways, and Gene Clusters in Plants. Plant Physiol..

[18] Nützmann H.W., Scazzocchio C., Osbourn A. (2018). Metabolic Gene Clusters in Eukaryotes. Annu. Rev. Genet..

[19] Nützmann H.W., Huang A., Osbourn A. (2016). Plant metabolic clusters-from genetics to genomics. New. Phytol..

[20] Lo Y.T., Shaw P.C. (2019). Application of next-generation sequencing for the identification of herbal products. Biotechnol. Adv..

[21] Courdavault V., O’Connor S.E., Oudin A., Besseau S., Papon N. (2020). Towards the Microbial Production of Plant-Derived Anticancer Drugs. Trends Cancer.

[22] Burlat V., Papon N., Courdavault V. (2023). Medicinal plants enter the single-cell multi-omics era. Trends Plant Sci..

[23] Chen C., Zhang X., Yue M. (2024). Spatial multi-omics in medicinal plants: From biosynthesis pathways to industrial applications. Trends Plant Sci..

[24] Li G., Cai M., Liao X., Ma G.L., Fan X. (2026). Exploring specialized metabolic pathways in medicinal plants with single-cell and spatial omics. Acta Pharm. Sin. B.

[25] Yamamoto K., Takahashi K., Mizuno H., Anegawa A., Ishizaki K., Fukaki H., Ohnishi M., Yamazaki M., Masujima T., Mimura T. (2016). Cell-specific localization of alkaloids in Catharanthus roseus stem tissue measured with Imaging MS and Single-cell MS. Proc. Natl. Acad. Sci. USA.

[26] Boughton B.A., Thinagaran D., Sarabia D., Bacic A., Roessner U. (2016). Mass spectrometry imaging for plant biology: A review. Phytochem. Rev..

[27] Bjarnholt N., Li B., D’Alvise J., Janfelt C. (2014). Mass spectrometry imaging of plant metabolites--principles and possibilities. Nat. Prod. Rep..

[28] Dong Y., Li B., Aharoni A. (2016). More than Pictures: When MS Imaging Meets Histology. Trends Plant Sci..

[29] St-Pierre B., Vazquez-Flota F.A., De Luca V. (1999). Multicellular compartmentation of catharanthus roseus alkaloid biosynthesis predicts intercellular translocation of a pathway intermediate. Plant Cell.

[30] Burlat V., Oudin A., Courtois M., Rideau M., St-Pierre B. (2004). Co-expression of three MEP pathway genes and geraniol 10-hydroxylase in internal phloem parenchyma of Catharanthus roseus implicates multicellular translocation of intermediates during the biosynthesis of monoterpene indole alkaloids and isoprenoid-derived primary metabolites. Plant J..

[31] Mahroug S., Courdavault V., Thiersault M., St-Pierre B., Burlat V. (2006). Epidermis is a pivotal site of at least four secondary metabolic pathways in Catharanthus roseus aerial organs. Planta.

[32] Miettinen K., Dong L., Navrot N., Schneider T., Burlat V., Pollier J., Woittiez L., van der Krol S., Lugan R., Ilc T. (2014). The seco-iridoid pathway from Catharanthus roseus. Nat. Commun..

[33] Larsen B., Fuller V.L., Pollier J., Van Moerkercke A., Schweizer F., Payne R., Colinas M., O’Connor S.E., Goossens A., Halkier B.A. (2017). Identification of Iridoid Glucoside Transporters in Catharanthus roseus. Plant Cell Physiol..

[34] Olsson M.E., Olofsson L.M., Lindahl A.L., Lundgren A., Brodelius M., Brodelius P.E. (2009). Localization of enzymes of artemisinin biosynthesis to the apical cells of glandular secretory trichomes of *Artemisia annua* L.. Phytochemistry.

[35] Olofsson L., Engström A., Lundgren A., Brodelius P.E. (2011). Relative expression of genes of terpene metabolism in different tissues of *Artemisia annua* L.. BMC Plant Biol..

[36] Tellez M.R., Canel C., Rimando A.M., Duke S.O. (1999). Differential accumulation of isoprenoids in glanded and glandless *Artemisia annua* L.. Phytochemistry.

[37] Bird D.A., Franceschi V.R., Facchini P.J. (2003). A tale of three cell types: Alkaloid biosynthesis is localized to sieve elements in opium poppy. Plant Cell.

[38] Weid M., Ziegler J., Kutchan T.M. (2004). The roles of latex and the vascular bundle in morphine biosynthesis in the opium poppy, Papaver somniferum. Proc. Natl. Acad. Sci. USA.

[39] Wang T., Wang F., Deng S., Wang K., Feng D., Xu F., Guo W., Yu J., Wu Y., Wuriyanghan H. (2025). Single-cell transcriptomes reveal spatiotemporal heat stress response in maize roots. Nat. Commun..

[40] Jiang B.P., Si J.Y., Luo H.M., Le L. (2025). Single-cell omics reveal the mechanisms of traditional Chinese medicines. Phytomedicine.

[41] Efroni I., Birnbaum K.D. (2016). The potential of single-cell profiling in plants. Genome Biol..

[42] Farmer A., Thibivilliers S., Ryu K.H., Schiefelbein J., Libault M. (2021). Single-nucleus RNA and ATAC sequencing reveals the impact of chromatin accessibility on gene expression in Arabidopsis roots at the single-cell level. Mol. Plant.

[43] Yamamoto K., Takahashi K., Caputi L., Mizuno H., Rodriguez-Lopez C.E., Iwasaki T., Ishizaki K., Fukaki H., Ohnishi M., Yamazaki M. (2019). The complexity of intercellular localisation of alkaloids revealed by single-cell metabolomics. New Phytol..

[44] Sun S., Shen X., Li Y., Li Y., Wang S., Li R., Zhang H., Shen G., Guo B., Wei J. (2023). Single-cell RNA sequencing provides a high-resolution roadmap for understanding the multicellular compartmentation of specialized metabolism. Nat. Plants.

[45] Vu A.H., Kang M., Wurlitzer J., Heinicke S., Li C., Wood J.C., Grabe V., Buell C.R., Caputi L., O’Connor S.E. (2024). Quantitative Single-Cell Mass Spectrometry Provides a Highly Resolved Analysis of Natural Product Biosynthesis Partitioning in Plants. J. Am. Chem. Soc..

[46] Mao X., Xia D., Xu M., Gao Y., Tong L., Lu C., Li W., Xie R., Liu Q., Jiang D. (2025). Single-Cell Simultaneous Metabolome and Transcriptome Profiling Revealing Metabolite-Gene Correlation Network. Adv. Sci..

[47] Li C., Wood J.C., Vu A.H., Hamilton J.P., Rodriguez Lopez C.E., Payne R.M.E., Serna Guerrero D.A., Gase K., Yamamoto K., Vaillancourt B. (2023). Single-cell multi-omics in the medicinal plant Catharanthus roseus. Nat. Chem. Biol..

[48] Li C., Colinas M., Wood J.C., Vaillancourt B., Hamilton J.P., Jones S.L., Caputi L., O’Connor S.E., Buell C.R. (2025). Cell-type-aware regulatory landscapes governing monoterpene indole alkaloid biosynthesis in the medicinal plant Catharanthus roseus. New Phytol..

[49] Marand A.P., Chen Z., Gallavotti A., Schmitz R.J. (2021). A cis-regulatory atlas in maize at single-cell resolution. Cell.

[50] Wang F., Wang F., Wu Y., Pu L., Le L. (2026). Single-cell insights into plant growth, adaptation, and evolution. J. Integr. Plant Biol..

[51] Kompauer M., Heiles S., Spengler B. (2017). Atmospheric pressure MALDI mass spectrometry imaging of tissues and cells at 1.4-μm lateral resolution. Nat. Methods.

[52] Spraker J.E., Luu G.T., Sanchez L.M. (2020). Imaging mass spectrometry for natural products discovery: A review of ionization methods. Nat. Prod. Rep..

[53] Dong Y., Li B., Malitsky S., Rogachev I., Aharoni A., Kaftan F., Svatoš A., Franceschi P. (2016). Sample Preparation for Mass Spectrometry Imaging of Plant Tissues: A Review. Front. Plant Sci..

[54] Chen A., Liao S., Cheng M., Ma K., Wu L., Lai Y., Qiu X., Yang J., Xu J., Hao S. (2022). Spatiotemporal transcriptomic atlas of mouse organogenesis using DNA nanoball-patterned arrays. Cell.

[55] McClune C.J., Liu J.C., Wick C., De La Peña R., Lange B.M., Fordyce P.M., Sattely E.S. (2025). Discovery of FoTO1 and Taxol genes enables biosynthesis of baccatin III. Nature.

[56] Jennewein S., Croteau R. (2001). Taxol: Biosynthesis, molecular genetics, and biotechnological applications. Appl. Microbiol. Biotechnol..

[57] Ajikumar P.K., Xiao W.H., Tyo K.E., Wang Y., Simeon F., Leonard E., Mucha O., Phon T.H., Pfeifer B., Stephanopoulos G. (2010). Isoprenoid pathway optimization for Taxol precursor overproduction in *Escherichia coli*. Science.

[58] Wu S., Morotti A.L.M., Yang J., Wang E., Tatsis E.C. (2024). Single-cell RNA sequencing facilitates the elucidation of the complete biosynthesis of the antidepressant hyperforin in St. John’s wort. Mol. Plant.

[59] Wang R., Zhang L., Li X., Sun W., Wang Y., Huang L., Wang J., Gao W., Guo L. (2025). Single-cell sequencing and mass spectrometry imaging reveal the multicellular compartmentalization map of plant triterpenes: A ginsenoside in Panax ginseng example. Plant Biotechnol. J..

[60] Yang L., Yang Z., Liu M., Wang S., Wu H., Yang Q., Huang L., Yang Y., Cui X., Liu Y. (2025). Integrated single-cell transcriptomics and spatial metabolomics unveil cellular differentiation and ginsenosides biosynthesis in Panax root tips. Hortic. Res..

[61] Zhang M., Li M., An Y., Liu C., Zhao Q., Zhang D., Pan B., Tan H. (2025). Single-nucleus transcriptomics reveal the morphogenesis and artemisinin biosynthesis in *Artemisia annua* glandular trichomes. Nat. Commun..

[62] Zhou P., Chen H., Dang J., Shi Z., Shao Y., Liu C., Fan L., Wu Q. (2022). Single-cell transcriptome of Nepeta tenuifolia leaves reveal differentiation trajectories in glandular trichomes. Front. Plant Sci..

[63] Liu L., Gao L., Wang Z.Y., Liu Y., Ju H., Lin H., Shi Z., Cai W.J., Li X., Huang Y.B. (2026). Cross-stage single-cell and spatial metabolome analyses reveal periderm specialization and tanshinone biosynthesis in *Salvia miltiorrhiza* roots. New Phytol..

[64] Hao X., Yang Y., Chen T., Fan X., Peng Y., Ruan Q., Zhou Q., Liu F., He J., Li Y. (2026). Cell-Specific Expression and Cellular Compartmental Regulation in Camptothecin Biosynthesis. Plant Biotechnol. J..

[65] Wang S., Zhang C., Li Y., Li R., Du K., Sun C., Shen X., Guo B. (2024). ScRNA-seq reveals the spatiotemporal distribution of camptothecin pathway and transposon activity in *Camptotheca acuminata* shoot apexes and leaves. Physiol. Plant..

[66] Zhang X., Miao Y., Xing Y., Zhang G., Zhang M., Thorogood C.J., Chen S., Huang L. (2026). Genome and Single-Cell Transcriptome Reveal the Evolution of Holoparasitic Plants: A Case Study of *Cistanche deserticola*. Plant Biotechnol. J..

[67] Zeng H., Shi M., Chen Z., Sun X., Zhang H., Huang Y., Chen Y., Ren J., Huang H., Borjigidai A. (2026). Mass Spectrometry Imaging Combined With Single-Cell Transcriptional Profiling Reveals the Multidimensional Spatial Distributions and Biosynthetic Pathways of Medicinal Components in *Andrographis paniculata*. Plant Biotechnol. J..

[68] Li R., Du K., Zhang C., Shen X., Yun L., Wang S., Li Z., Sun Z., Wei J., Li Y. (2024). Single-cell transcriptome profiling reveals the spatiotemporal distribution of triterpenoid saponin biosynthesis and transposable element activity in *Gynostemma pentaphyllum* shoot apexes and leaves. Front. Plant Sci..

[69] Wang X., Ge X., Zhong W., Wu D., Yang Z., Zhan R., Chen L., Li H. (2025). Construction of a single-cell transcriptome atlas for Pogostemon cablin embryoids reveals PcNAC048 as a dual regulator coordinating lateral root morphogenesis and patchouli alcohol biosynthesis. Commun. Biol..

[70] Wang J., Zhou Y., Zhang M., Li X., Liu T., Liu Y., Xie H., Wang K., Li P., Xu Z. (2025). Resolving floral development dynamics using genome and single-cell temporal transcriptome of *Dendrobium devonianum*. Plant Biotechnol. J..

[71] Page M.J., McKenzie J.E., Bossuyt P.M., Boutron I., Hoffmann T.C., Mulrow C.D., Shamseer L., Tetzlaff J.M., Akl E.A., Brennan S.E. (2021). The PRISMA 2020 statement: An updated guideline for reporting systematic reviews. BMJ.

[72] Ryu K.H., Huang L., Kang H.M., Schiefelbein J. (2019). Single-Cell RNA Sequencing Resolves Molecular Relationships Among Individual Plant Cells. Plant Physiol..

[73] Jean-Baptiste K., McFaline-Figueroa J.L., Alexandre C.M., Dorrity M.W., Saunders L., Bubb K.L., Trapnell C., Fields S., Queitsch C., Cuperus J.T. (2019). Dynamics of Gene Expression in Single Root Cells of *Arabidopsis thaliana*. Plant Cell.

[74] Payne R.M., Xu D., Foureau E., Teto Carqueijeiro M.I., Oudin A., Bernonville T.D., Novak V., Burow M., Olsen C.E., Jones D.M. (2017). An NPF transporter exports a central monoterpene indole alkaloid intermediate from the vacuole. Nat. Plants.

[75] Dong S., Chen H., Sun S., Guo M., Sun C., Chen S., Luo H. (2026). Metabolomic and Single-Cell Transcriptomic Analyses Shed Light on Secondary Metabolite Profiling and Potential Developmental Dynamics of Glandular Trichomes in *Artemisia argyi*. Plant Biotechnol. J..

[76] Menke F.L., Champion A., Kijne J.W., Memelink J. (1999). A novel jasmonate- and elicitor-responsive element in the periwinkle secondary metabolite biosynthetic gene Str interacts with a jasmonate- and elicitor-inducible AP2-domain transcription factor, ORCA2. Embo J..

[77] Kerk N.M., Ceserani T., Tausta S.L., Sussex I.M., Nelson T.M. (2003). Laser capture microdissection of cells from plant tissues. Plant Physiol..

[78] Guillotin B., Rahni R., Passalacqua M., Mohammed M.A., Xu X., Raju S.K., Ramírez C.O., Jackson D., Groen S.C., Gillis J. (2023). A pan-grass transcriptome reveals patterns of cellular divergence in crops. Nature.

[79] Mo Y., Jiao Y. (2022). Advances and applications of single-cell omics technologies in plant research. Plant J..

[80] Lin J.L., Chen L., Wu W.K., Guo X.X., Yu C.H., Xu M., Nie G.B., Dun J.L., Li Y., Xu B. (2023). Single-cell RNA sequencing reveals a hierarchical transcriptional regulatory network of terpenoid biosynthesis in cotton secretory glandular cells. Mol. Plant.

[81] Maher K.A., Bajic M., Kajala K., Reynoso M., Pauluzzi G., West D.A., Zumstein K., Woodhouse M., Bubb K., Dorrity M.W. (2018). Profiling of Accessible Chromatin Regions across Multiple Plant Species and Cell Types Reveals Common Gene Regulatory Principles and New Control Modules. Plant Cell.

[82] Lu Z., Marand A.P., Ricci W.A., Ethridge C.L., Zhang X., Schmitz R.J. (2019). The prevalence, evolution and chromatin signatures of plant regulatory elements. Nat. Plants.

[83] Liu Q., Ma W., Chen R.Y., Li S.T., Wang Q.F., Wei C., Hong Y.G., Sun H.X., Cheng Q., Zhao J.J. (2024). Multiome in the Same Cell Reveals the Impact of Osmotic Stress on Arabidopsis Root Tip Development at Single-Cell Level. Adv. Sci..

[84] Dueñas M.E., Klein A.T., Alexander L.E., Yandeau-Nelson M.D., Nikolau B.J., Lee Y.J. (2017). High spatial resolution mass spectrometry imaging reveals the genetically programmed, developmental modification of the distribution of thylakoid membrane lipids among individual cells of maize leaf. Plant J..

[85] Shroff R., Vergara F., Muck A., Svatos A., Gershenzon J. (2008). Nonuniform distribution of glucosinolates in *Arabidopsis thaliana* leaves has important consequences for plant defense. Proc. Natl. Acad. Sci. USA.

[86] Zhan X., Qiu T., Zhang H., Hou K., Liang X., Chen C., Wang Z., Wu Q., Wang X., Li X.L. (2023). Mass spectrometry imaging and single-cell transcriptional profiling reveal the tissue-specific regulation of bioactive ingredient biosynthesis in Taxus leaves. Plant Commun..

[87] Yu C., Hou K., Zhang H., Liang X., Chen C., Wang Z., Wu Q., Chen G., He J., Bai E. (2023). Integrated mass spectrometry imaging and single-cell transcriptome atlas strategies provide novel insights into taxoid biosynthesis and transport in *Taxus mairei* stems. Plant J..

[88] Bui V.H., Wood J.C., Vaillancourt B., Hamilton J.P., Carlton L.H., Dang T.T., Buell C.R., Li C. (2026). Single Cell Multi-Omics Reveals Rare Biosynthetic Cell Types in the Medicinal Tree *Camptotheca acuminata*. Plant Biotechnol. J..

[89] Shen Q., Zhang L., Liao Z., Wang S., Yan T., Shi P., Liu M., Fu X., Pan Q., Wang Y. (2018). The Genome of *Artemisia annua* Provides Insight into the Evolution of Asteraceae Family and Artemisinin Biosynthesis. Mol. Plant.

[90] Qin Z., Chen C., Zhang T., Wu Y., Zheng Y. (2025). Single-cell RNA sequencing reveals transcriptional regulation and metabolic pathways of terpenoid biosynthesis in developing *Cinnamomum camphora* leaf cells. Curr. Plant Biol..

[91] Zhao B., Gao Y., Ma Q., Wang X., Zhu J.K., Li W., Wang B., Yuan F. (2024). Global dynamics and cytokinin participation of salt gland development trajectory in recretohalophyte Limonium bicolor. Plant Physiol..

[92] Yu F., De Luca V. (2013). ATP-binding cassette transporter controls leaf surface secretion of anticancer drug components in Catharanthus roseus. Proc. Natl. Acad. Sci. USA.

[93] Gayoso A., Lopez R., Xing G., Boyeau P., Valiollah Pour Amiri V., Hong J., Wu K., Jayasuriya M., Mehlman E., Langevin M. (2022). A Python library for probabilistic analysis of single-cell omics data. Nat. Biotechnol..

[94] Kleshchevnikov V., Shmatko A., Dann E., Aivazidis A., King H.W., Li T., Elmentaite R., Lomakin A., Kedlian V., Gayoso A. (2022). Cell2location maps fine-grained cell types in spatial transcriptomics. Nat. Biotechnol..

[95] Biancalani T., Scalia G., Buffoni L., Avasthi R., Lu Z., Sanger A., Tokcan N., Vanderburg C.R., Segerstolpe Å., Zhang M. (2021). Deep learning and alignment of spatially resolved single-cell transcriptomes with Tangram. Nat. Methods.

[96] Wolny A., Cerrone L., Vijayan A., Tofanelli R., Barro A.V., Louveaux M., Wenzl C., Strauss S., Wilson-Sánchez D., Lymbouridou R. (2020). Accurate and versatile 3D segmentation of plant tissues at cellular resolution. Elife.

[97] Dührkop K., Fleischauer M., Ludwig M., Aksenov A.A., Melnik A.V., Meusel M., Dorrestein P.C., Rousu J., Böcker S. (2019). SIRIUS 4: A rapid tool for turning tandem mass spectra into metabolite structure information. Nat. Methods.

[98] Huber F., Ridder L., Verhoeven S., Spaaks J.H., Diblen F., Rogers S., van der Hooft J.J.J. (2021). Spec2Vec: Improved mass spectral similarity scoring through learning of structural relationships. PLoS Comput. Biol..

[99] Jumper J., Evans R., Pritzel A., Green T., Figurnov M., Ronneberger O., Tunyasuvunakool K., Bates R., Žídek A., Potapenko A. (2021). Highly accurate protein structure prediction with AlphaFold. Nature.

[100] Abramson J., Adler J., Dunger J., Evans R., Green T., Pritzel A., Ronneberger O., Willmore L., Ballard A.J., Bambrick J. (2024). Accurate structure prediction of biomolecular interactions with AlphaFold 3. Nature.

